# Development of a pediatric pig model of extracorporeal cardiopulmonary resuscitation-induced acute kidney injury

**DOI:** 10.1242/dmm.052726

**Published:** 2026-10-02

**Authors:** Lindsay M. Thomson, Christopher A. Mancuso, Robert Hyslop, Ludmila Khailova, Camille N. Chun, Jack Zakrzewski, Sierra S. Niemiec, Tanner M. Lehmann, Benjamin S. Frank, Jesse A. Davidson

**Affiliations:** ^1^ Department of Pediatrics, University of Colorado Anschutz Medical Campus, Aurora, CO 80045, USA; ^2^ Department of Biostatistics and Informatics, University of Colorado Anschutz Medical Campus, Aurora, CO, USA; ^3^ Deparment of Surgery, University of Colorado Anschutz Medical Campus, Aurora, CO 80045, USA

**Keywords:** Extracorporeal membrane oxygenation (ECMO), Cardiac arrest, Transcriptomics, Infant, Swine

## Abstract

Acute kidney injury (AKI) is a common and severe morbidity following extracorporeal cardiopulmonary resuscitation (ECPR), particularly in children, where AKI is associated with a fourfold increased risk of death. Research into the mechanisms and treatment of ECPR-induced AKI is hampered by the lack of relevant translational models. In this study, we developed a pediatric swine model of ECPR-induced AKI and defined the histological and transcriptomic changes seen in the kidney following ECPR. Four infant swine underwent hyperkalemic cardiac arrest for 5, 10, 15 or 20 min, followed by 4 h of extracorporeal membrane oxygenation. Mechanically ventilated animals (*n=*9) were used for comparison. ECPR animals demonstrated progressive histologic, biomarker and physiologic kidney injury with increasing cardiac arrest time. All ECPR-exposed animals demonstrated a distinct transcriptomic response compared to controls, with 1433 differentially expressed genes covering a range of biologic systems including oxidative stress, renal angiogenesis and solute channel formation, protein folding/misfolding, and ribosome biogenesis. This study demonstrates the ability to model ECPR-induced kidney injury in a titratable fashion, serving as a platform for mechanistic and therapeutic studies for this important morbidity.

## Teaser

**Summary:** This model of pediatric extracorporeal cardiopulmonary resuscitation/extracorporeal membrane oxygenation results in reproducible acute kidney injury with acute histologic, physiologic, biomarker and transcriptomic changes that are titratable based on the duration of cardiac arrest.

## INTRODUCTION

Use of extracorporeal membrane oxygenation (ECMO) for cardiorespiratory support is increasing worldwide, with over 20,000 ECMO runs and center members from over 50 countries [Extracorporeal Life Support Organization (ELSO), Extracorporeal life support (ECLS) International Summary of Statistics, accessed 17 October, 2023]. Pediatric ECMO constitutes ∼40% of total historic ECMO runs and is a growing cohort. Among pediatric ECMO cases (excluding the neonatal population), cardiac failure is the prevailing indication, and nearly one in five pediatric ECMO runs is initiated as part of extracorporeal cardiopulmonary resuscitation (ECPR) (ECLS International Summary of Statistics, accessed 17 October, 2023). While ECMO is life saving and life sustaining, it remains associated with significant morbidity. Acute kidney injury (AKI) occurs in up to 70% of ECMO runs (Foti et al., 2021). AKI during ECMO is an independent risk factor for mortality and is associated with up to a fourfold increase in death (Wu et al., 2008; Lin et al., 2006).

Factors leading to AKI are myriad: at the macroscopic level, patients often suffer poor perfusion and ischemia from the underlying disease that is exacerbated during ECMO by poorly pulsatile flow and hypoperfusion/reperfusion injury (Yan et al., 2025). At the metabolic and cellular levels, side effects from ECMO contributing to AKI include mechanical shear stress and cell activation from direct and prolonged contact with the ECMO circuit (Millar et al., 2016). This results in a global inflammatory response with activation of cellular and humoral cascades, including activation of complement, coagulation and fibrinolytic pathways, endotoxin release, cytokine production, and endothelial injury, all result in the production and release of oxygen (O_2_)-free radicals, nitric oxide, arachidonic acid derivatives and proteolytic enzymes (Thiagarajan et al., 2007; Bembea et al., 2020). These injurious pathways manifest in clinical kidney dysfunction, typically estimated at the bedside by using single biomarkers – most commonly creatinine, cystatin C and urine neutrophil gelatinase-associated lipocalin (LCN2, also known as NGAL – which are downstream surrogates of reduced glomerular filtration or tubular injury. However, single biomarker testing is insufficient to address the complicated reality of a body's response to multiorgan injury, particularly in settings of severe systemic dysregulation, such as ECMO.

Diagnostic approaches that allow for the simultaneous measurement of hundreds of biomarkers (‘omics’) could help overcome this problem. Omics allows for exploring candidate biomarkers for multiple morbidities, evaluating their interaction during injury and recovery, and determining therapeutic targets for translational development (Bujak et al., 2015; Wishart, 2016; Chong et al., 2018). A large animal model of ECMO-induced AKI can uniquely identify kidney dysfunction at the whole organ, tissue histology and molecular levels, providing a comprehensive platform for study. Here, we described a model development study that establishes a pediatric pig model of AKI on ECMO after cardiac arrest (ECPR) and validates the model by using clinically available injury assessment methods, including histology, urinalysis, NGAL, creatinine and blood urea nitrogen. We additionally compared traditional clinical evidence of organ injury with RNA sequencing (RNAseq) to understand the kidney-specific transcriptome after ECPR.

## RESULTS

### Model development and characterization

Four pigs underwent ECPR were successfully cannulated and supported on ECMO for 4 h following variable periods of cardiac arrest. All four pigs experienced cardiac arrest with a period of asystole. Two out of four pigs (warm ischemic times – i.e. arrest time – 10 and 20 min) remained asystolic for the duration of the ECMO period. Pigs with a warm ischemic time of 5 and 15 min regained inconsistent electrical activity with heart rates ranging from 34−70 beats per min by 3 h of ECMO support. The pulse pressure generated by electrical activity was ∼30 mm mercury (mmHg) in the 5-min arrest pig and 16–19 mmHg in the 15-min arrest pig. Electrolytes and blood gases were monitored post intubation and shortly before cardiac arrest, followed by hourly monitoring while on ECMO. The average arterial pH prior to arrest was within a normal range at 7.44±0.07, and the average pH while on ECMO was mildly acidotic at 7.30±0.04 (*P*=0.048) due to lactic acidosis (average lactate 7.1±1.7 mmol/l) in the setting of normal or low partial pressure of carbon dioxide (average pCO_2_ 38.5±3.8 mmHg) (Table 1).

**Table 1.** Summary of average chemistry values from arterial blood samples for each intervention.

Intervention	pH (±s.d.)	pCO_2_ (mmHg)	HCO_3_^−^ (mEq/l)	Na^+^ (mEq/l)	K^+^ (mEq/l)	Glucose (mg/dl)	Ca^2+^ (mmol/l)	Lactate (mmol/l)
5 min arrest	7.32±0.05	40±2	20.8±1.3	132±1.6	>9	136±30	1.45±0.15	4.8±1.4
10 min arrest	7.34±0.06	33±3	18.2±1.4	135±0.8	>9	205±26	1.31±0.13	6.9±1.5
15 min arrest	7.28±0.08	39±7	18.0±0.5	137±0.8	>9	185±31	1.36±0.19	8.5±0.2
20 min arrest	7.27±0.03	42±2	19.3±1.3	135±1.5	>9	345±60	1.34±0.19	8.3±2.2
Controls averaged	7.41	43	26.70	137	4.6	89	1.38	0.73
Controls SD	0.05	5	2.30	2	0.4	32	0.12	0.34

In ECMO pigs (*n=*4), the average represents the average value plus the standard deviation from hourly whole blood samples while supported on ECMO from immediately post-arrest to immediately pre-euthanasia timepoints. Those pigs also experienced increasing hyperglycemia. ECMO pigs suffered from severe lactic acidosis, most severe in the 15- and 20-min arrest pigs. CO_2_ was titrated to normal values via sweep gas on the ECMO circuit. All pigs suffered marked hyperkalemia above the detection level in our assay. For controls (*n=*9), the average represents the average plus the standard deviation of all hourly samples for all mechanically ventilated control pigs. Control pigs maintained a normal pH with no lactatemia and no measured electrolyte disturbances. s.d., standard deviation; mEq, milliequivalent; mmHg, pressure unit equal to that exerted under standard gravity by a height of 1 mm of mercury; pCO_2_, partial pressure of carbon dioxide (i.e. pressure of CO_2_ within arterial blood).

All four pigs experienced multiple electrolyte derangements on ECMO, including significant hyperkalemia above the level of assay detection (K>9 in all pigs) and hyperglycemia (averaging 218±90 mg/dl), i.e. high potassium ions (K^+^) and glucose levels, respectively, in the blood. Serum levels of sodium (Na^+^) and ionized calcium (Ca^2+^) were normal, i.e. averaging 135±2 milliequivalent (mEq)/l and 1.4±0.1 mmol/l, respectively (Table 1). All nine control animals were successfully supported with mechanical ventilation for a period of 8 h. Control pigs maintained a normal pH with normal lactate levels and no appreciable electrolyte disturbances (Table 1).

### Histology

All piglets that had experienced cardiac arrest and 4 h on ECMO (*n*=4) demonstrated early evidence of kidney injury by histologic evaluation. All piglets demonstrated some level of renal tubular dilation and epithelial flattening (*n*=4), with three demonstrating bleb formation (i.e. apical epithelial protrusion) and one (after a 20-min arrest) cast formation (i.e. intraluminal accumulation of proteinaceous/cellular material) (Fig. 1A). The amount of injury worsened with the duration of cardiac arrest, with animals experiencing 15- and 20-min arrests demonstrating more widespread histologic changes. Those animals had an injury score of ‘3’ or ‘4’, indicating that >50% or 75%, respectively, of the viewed sample was injured. In contrast, control animals demonstrated evidence of injury in only seven out of 27 kidney sections, distributed across six animals. Five of the seven sections with injury demonstrated minimal injury (grade 1 tubular dilatation/epithelial flattening), and two sections showed grade 2 changes (Table 2). No control animals demonstrated evidence of higher degree injury, neither blebbing nor cortical/medullary casts (Fig. 1B).

**Fig. 1.**
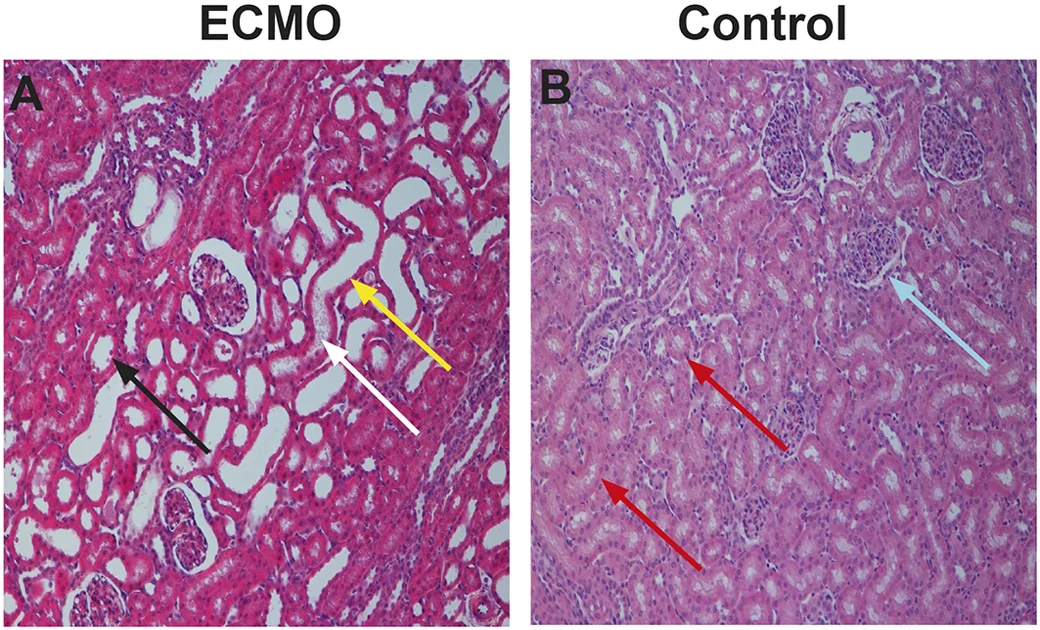
**Comparison of kidney histology in ECMO and healthy control piglets.** (A,B) Histologic evaluation of kidney injury by using validated scoring ranging between grade 0 and 4 (with 0 indicating the least and 4 indicating the greatest degree of injury). Changes of moderate to severe acute kidney injury (A) are seen in ECMO animals (*n=*4), including tubular dilation and epithelial thinning (yellow arrow), epithelial bleb formation (black arrow) and early cast formation (white arrow). By contrast, kidneys of control animals (B) show a grade of 0, with healthy glomeruli (blue arrow) and proximal tubules in cross section and longitudinal sections (red arrows).

**Table 2.** Degree of histologic kidney injury scored in ECMO and control piglets

Treatment	Animal condition	Sample (*n*=3 per animal)	Low power tubular dilatation or epithelial flattening	Bleb formation	Change in creatinine (mg/dl)	Change in urine NGAL (ng/ml)	Change in proteinuria (mg/dl)	Urine output (ml/kg/h)
**ECMO**	**ECMO 1 (5 min)**	**1**	0	n.o.	0	+309	0	4.2
	**ECMO 1 (5 min)**	**2**	0	n.o.				
	**ECMO 1 (5 min)**	**3**	1	n.o.				
	**ECMO 2 (10 min)**	**1**	0	n.o.	+0.1	+357	1985	1.8
	**ECMO 2 (10 min)**	**2**	1	yes				
	**ECMO 2 (10 min)**	**3**	0	n.o.				
	**ECMO 3 (15 min)**	**1**	2	yes	0	+335	0	5.0
	**ECMO 3 (15 min)**	**2**	4	yes				
	**ECMO 3 (15 min)**	**3**	1	yes				
	**ECMO 4 (20 min)**	**1**	2	yes	+0.1	+313	70	0.8
	**ECMO 4 (20 min)**	**2**	3	yes				
	**ECMO 4 (20 min)**	**3**	3	yes				
**Mechanical ventilation control**	**Control 1**	**1**	1	n.o.	−0.2	−186	−15	0.5
	**Control 1**	**2**	1	n.o.				
	**Control 1**	**3**	0	n.o.				
	**Control 2**	**1**	0	n.o.	−0.2	−137	n.a.	2.2
	**Control 2**	**2**	1	n.o.				
	**Control 2**	**3**	0	n.o.				
	**Control 3**	**1**	0	n.o.	−0.2	−370	15	0.8
	**Control 3**	**2**	0	n.o.				
	**Control 3**	**3**	0	n.o.				
	**Control 4**	**1**	0	n.o.	0	−370	n.a.	1.6
	**Control 4**	**2**	2	n.o.				
	**Control 4**	**3**	0	n.o.				
	**Control 5**	**1**	2	n.o.	0	n.a.	−15	0.5
	**Control 5**	**2**	0	n.o.				
	**Control 5**	**3**	0	n.o.				
	**Control 6**	**1**	0	n.o.	−0.4	−109	0	n.a.
	**Control 6**	**2**	0	n.o.				
	**Control 6**	**3**	0	n.o.				
	**Control 7**	**1**	0	n.o.	−0.1	−134	−15	1.4
	**Control 7**	**2**	1	n.o.				
	**Control 7**	**3**	0	n.o.				
	**Control 8**	**1**	1	n.o.	−0.1	−180	−15	2.7
	**Control 8**	**2**	0	n.o.				
	**Control 8**	**3**	0	n.o.				
	**Control 9**	**1**	0	n.o.	−0.1	−65	n.a.	1.1
	**Control 9**	**2**	0	n.o.				
	**Control 9**	**3**	0	n.o.				

Individual sections (*n*=3 for each animal) for histologic evaluation of kidney samples are shown for ECMO pigs (*n=*4) (organized by arrest condition) and each control animal (*n=*9). Control animals demonstrated minimal histologic evidence of kidney injury, and control animals showed either no change or a decrease in creatinine and urine NGAL, and proteinuria, probably due to intravenous hydration. 0, zero change from pre-ECMO to end-ECMO urine or blood samples. n.o., data not observed.; n.a., data not available due to sample unavailability or urinalysis sample error.

### Clinical biomarkers of AKI

Two out of four ECMO animals (10 and 20 min of arrest) experienced traditional clinical evidence of AKI marked by low urine output while on ECMO (<1 ml/kg/h), proteinuria and a small early rise in creatinine levels (Fig. 2A). Each AKI pig had an early creatinine level increase of 0.1 mg/dl and an increase in proteinuria from pre- to post-ECMO urinalyses. The pig with the most significant increase in proteinuria was that with the 10-min arrest, whose urine protein increased from 15 to 2000 mg/dl. The pig with a 20-min arrest had a more-modest urine protein increase from 30 to 100 mg/dl. In contrast, pigs with robust urine output and no increase in creatinine (5 and 15 min of arrest) exhibited similarly low levels of proteinuria pre- and post-ECMO (Fig. 2A and Table 2). Interestingly, those animals were the only ones to demonstrate visibly bloody urine with 5−10 blood/hemolysis on post-ECMO urinalysis. However, this did not correlate with microscopic urinalysis that did not identify abnormalities in any pig, i.e. no changes in white or red blood cell counts, calcium oxalate (CaC_2_O_4_) crystals, struvite crystals (NH_4_MgPO_4_**·**H_2_O) or hyaline casts, overall suggesting the presence of hemolysis with hemoglobinuria. Similarly, control pigs showed either no change or a decrease in creatinine and showed either no change in urine protein or decreased proteinuria by the end of the experimental period (Table 2).

**Fig. 2.**
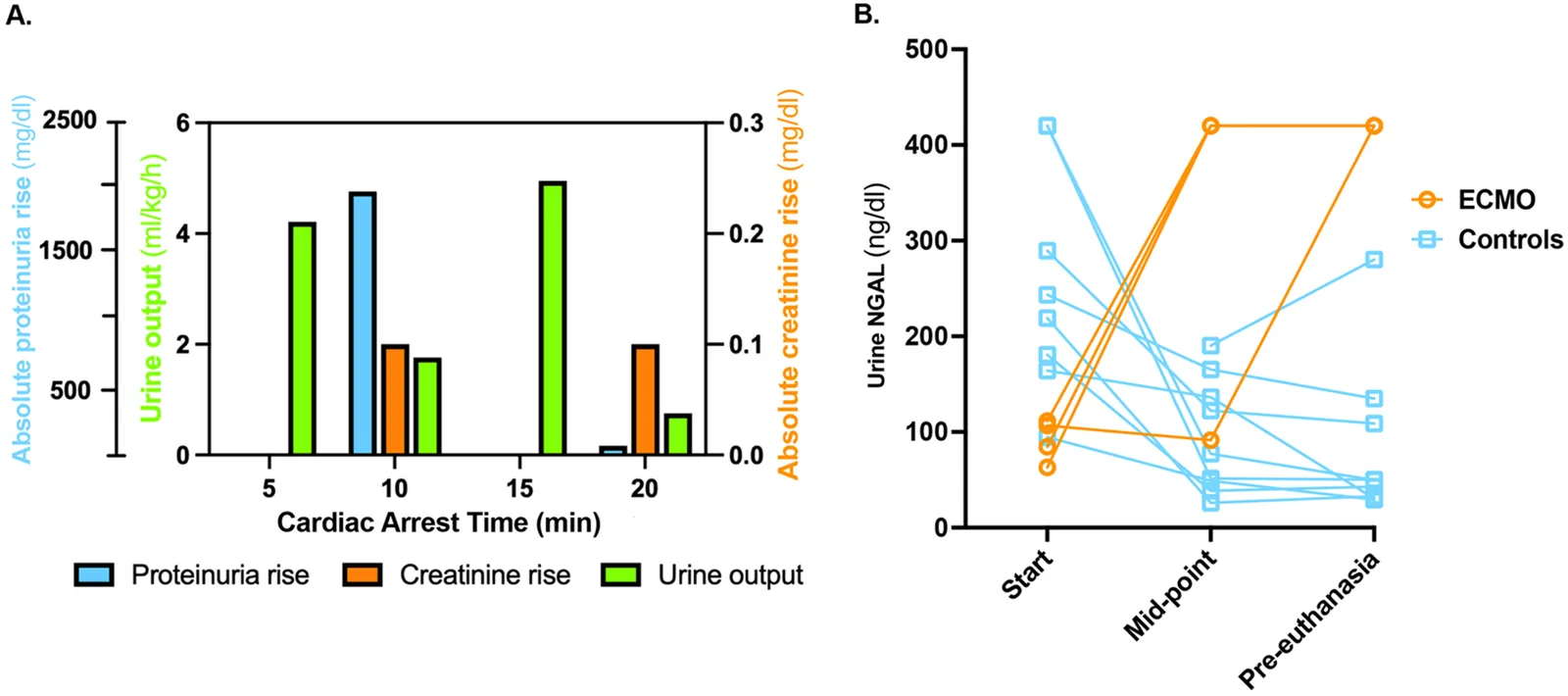
**Clinical markers of kidney injury at multiple time points in ECMO piglets, compared to controls.** (A) Bar graphs showing the rise in proteinuria (blue) and creatinine (orange) from pre-arrest levels until the end of ECMO, as well as the average urine output (green) per hour over the period of ECMO (*n*=4 piglets for all). During ECMO, two piglets experienced a severe reduction (<1 ml/kg/h) in urine output and a substantial increase (0.1 mg/dl) in proteinuria (blue) and creatinine (orange) levels compared with output/levels pre cardiac arrest and after ECMO. (B) Plotted are levels of urine NGAL, a more modern clinical marker of kidney injury in ECMO (orange, *n=*4) and control (blue, *n=*9) piglets. Over time, NGAL levels increased in ECMO animals, whereas levels decreased in all but one control piglets. Notice that urine for time point 0 was unavailable for one control piglet. Collectively, these data suggest early evidence of kidney injury in ECMO piglets.

We also tested urine for NGAL, a more modern marker of AKI. Many animals, including ECMO and control pigs (controls>ECMO), had elevated urine NGAL at the start of the experiment in the setting of pre-procedural fasting. All four ECMO pigs demonstrated substantial increases from the start of the experiment to pre-euthanasia, with pre-euthanasia values being statistically significantly increased compared with those of controls (*P*<0.005) (Fig. 2B and Table 2). Three of four pigs (5, 15 and 20 min of arrest) reached the maximum assay detection level by the experimental mid-point and sustained this maximal level to the post-euthanasia sample; the 10-min arrest pig achieved the maximum detection level by the time of euthanasia. In contrast to ECMO pigs, mechanically ventilated control pigs all experienced recovery with a decrease of urine NGAL throughout the experiment.

### Global pattern of gene expression after exposure to ECPR

To evaluate the global transcriptomic response to ECPR, we first performed an unsupervised dimension reduction analysis, i.e. principal component analysis (PCA). ECPR-exposed animals could be clearly distinguished from controls by their complete transcriptomic profile (Fig. 3). Animals with the shortest cardiac arrest times (i.e. 5 min and 10 min) and milder histologic injury were distinct from controls, while long arrest times (i.e. 15 min and 20 min) with greater histologic injury resulted in a further shift of the transcriptome away from that of the control animals. RNAseq identified a total of 1889 differentially expressed genes (DEGs), representing 6.9% of the RNA sequenced. Of these DEGs, 901 were significantly upregulated and 988 downregulated in the ECPR animals versus controls. The extent of significant up- and downregulated DEGs are shown in Fig. 4.

**Fig. 3.**
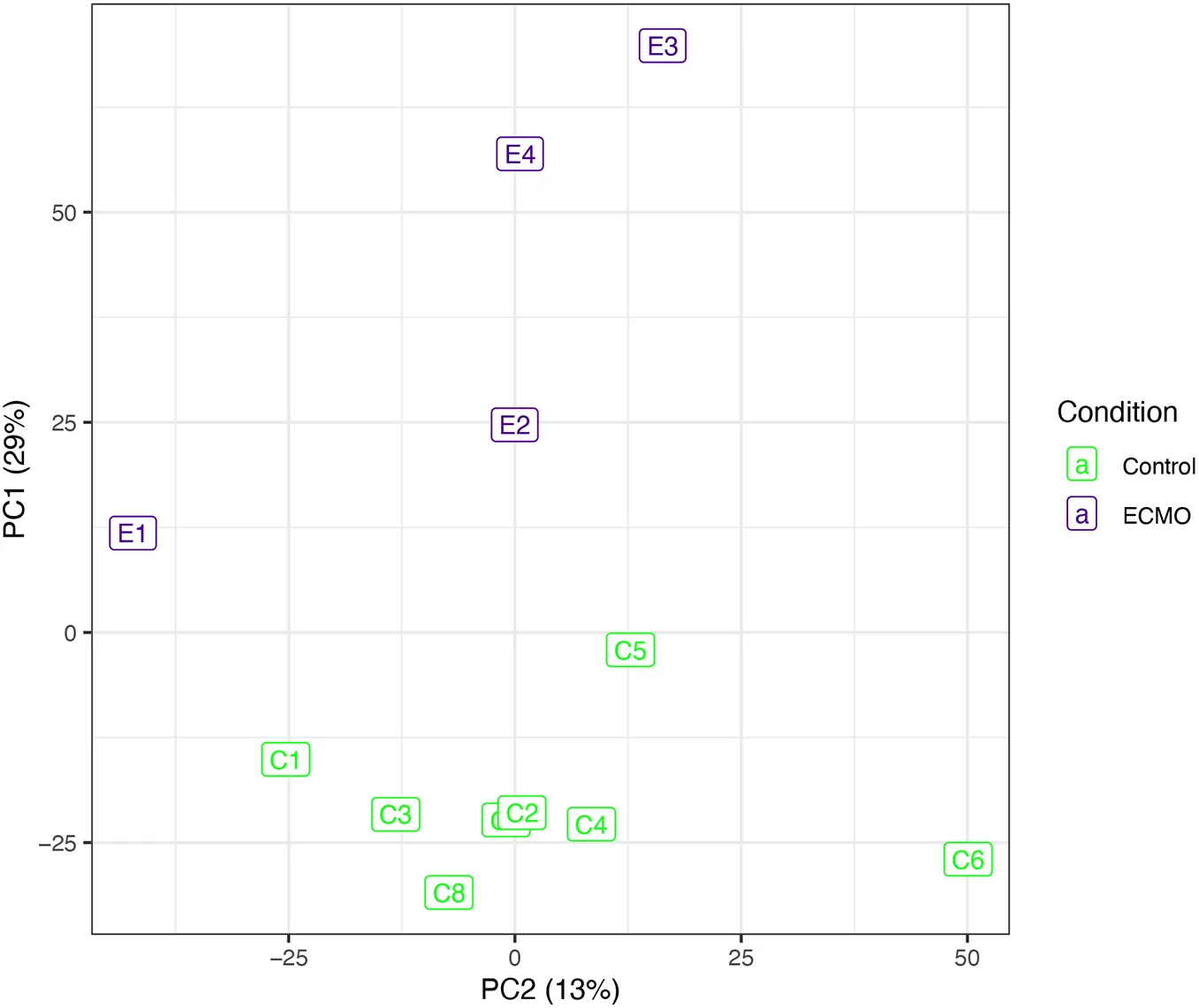
**Principal component analysis of ECMO compared to control animals.** Principal component analysis (PCA) of the global transcriptome of ECMO (purple, *n=*4) versus mechanically ventilated control (green, *n=*9) animals demonstrates clear differentiation in ECMO animals. The animal with the shortest duration of cardiac arrest (E1) demonstrated the least separation from the control animals. E1, 5 min cardiac arrest time; E2, 10 min cardiac arrest time; E3, 15 min cardiac arrest time; E4, 20 min cardiac arrest time; PC1, principal component of the PCA that describes the most variance; PC2, principal component of the PCA that describes the second-most variance in data, with the relative percentages of variance described reflected.

**Fig. 4.**
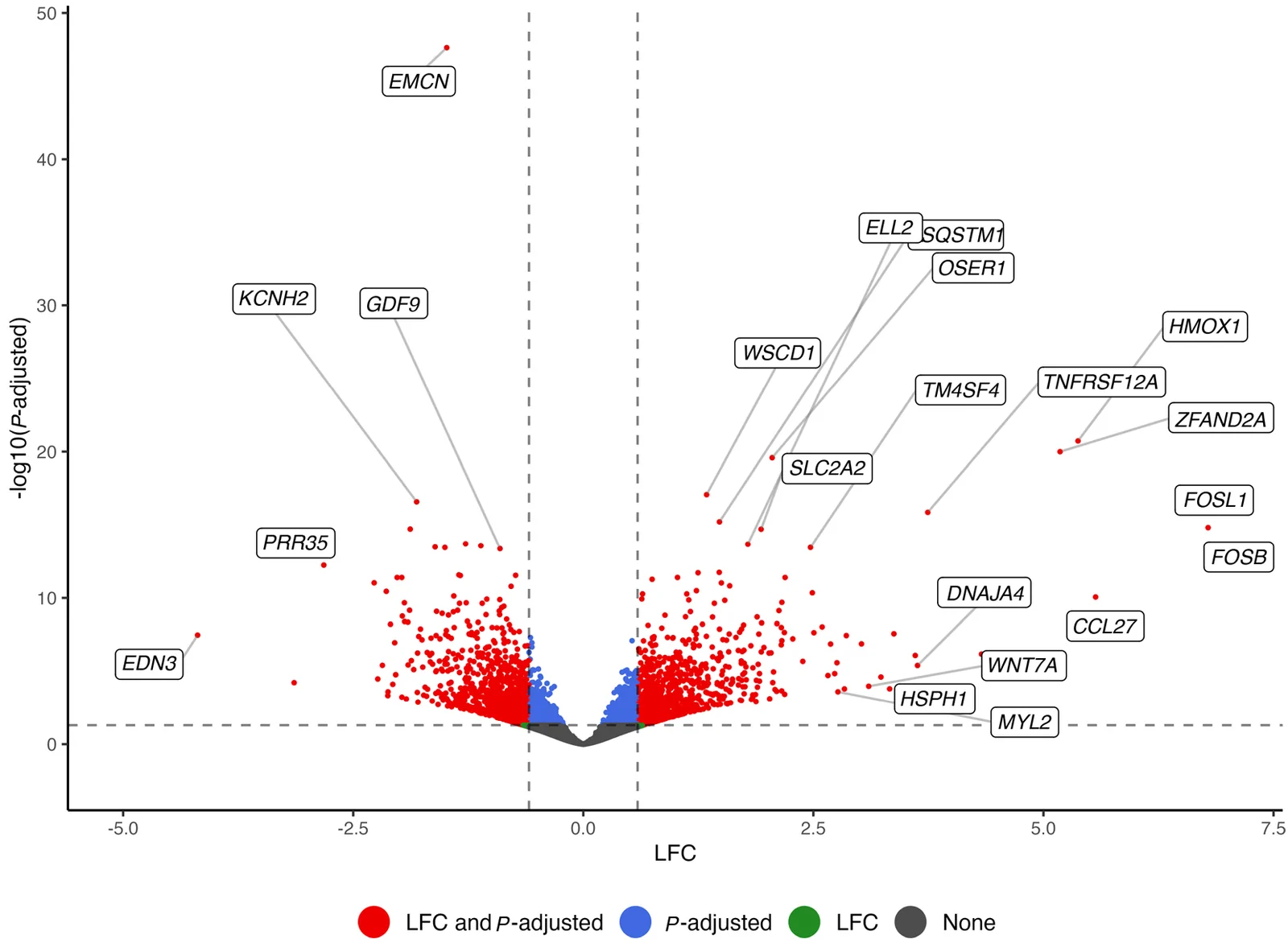
**Volcano plot of differentially expressed genes in ECPR animals compared to controls.** Differential expression analysis found 901 upregulated differentially expressed genes (DEGs) and 988 downregulated DEGs.

### Systems biology analysis: functional enrichment of up- or downregulated gene sets

Not all genes have human equivalents documented in the literature, so we focused on DEGs with pig-to-human orthologs for subsequent gene enrichment. Of those, we found 743 upregulated and 737 downregulated human gene equivalents. We performed gene set enrichment analysis to identify the most highly enriched biologic systems separately for up- and downregulated genes (Fig. 5). The key systems enriched in the upregulated genes were cytokine response, chemotaxis and protein misfolding response/endoplasmic reticulum stress. The key systems enriched in the downregulated genes were kidney and neuron morphogenesis, and cell-junction assembly.

**Fig. 5.**
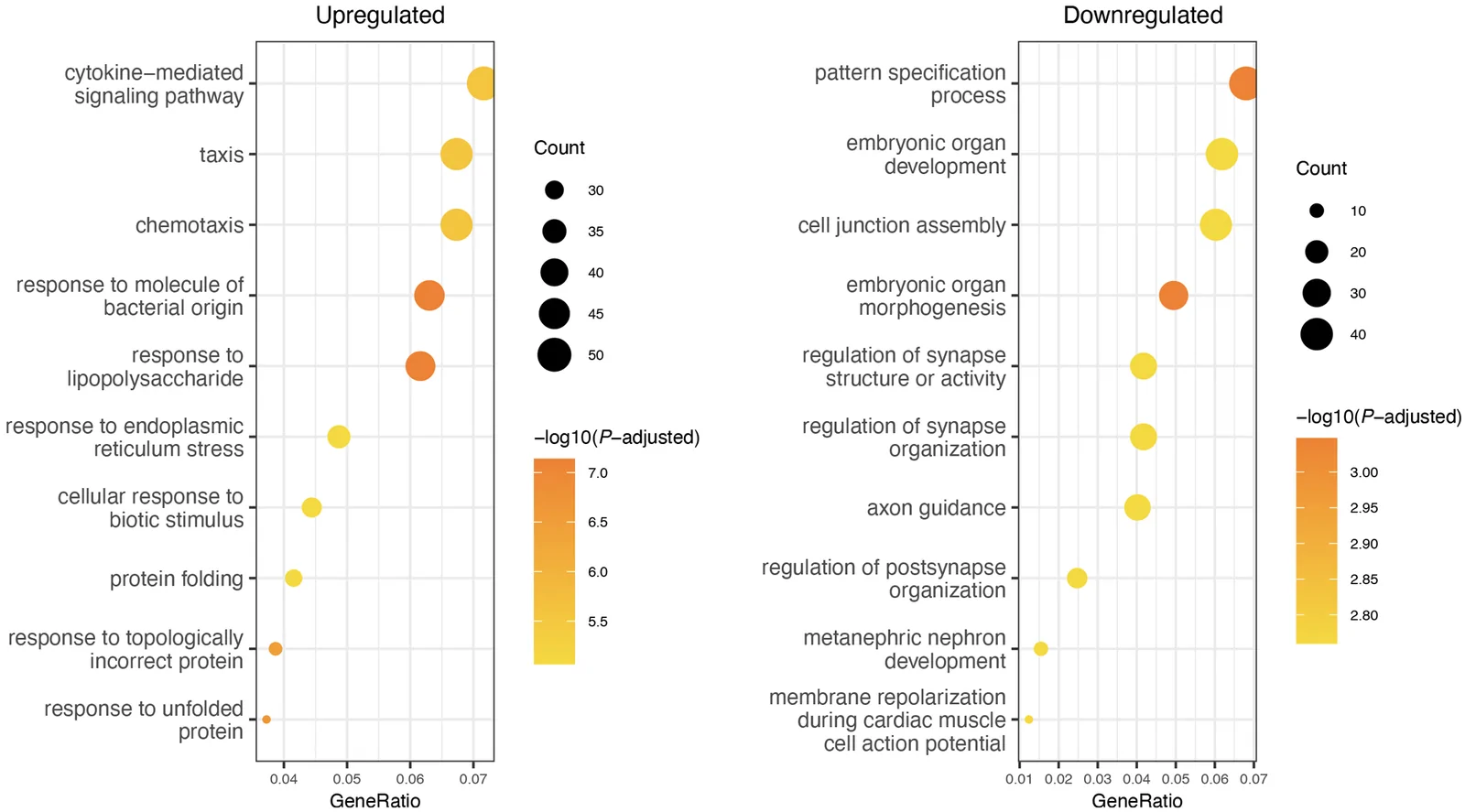
**Enriched biologic systems in up- and downregulated differentially expressed genes.** Left: Enriched biologic systems among the upregulated differentially expressed genes (DEGs). Right: Enriched biologic systems among the downregulated DEGs. Differential expression analysis found 901 upregulated differentially expressed genes (DEGs) and 988 downregulated DEGs. The key systems enriched in the upregulated genes were cytokine response, chemotaxis and protein misfolding response/endoplasmic reticulum stress. The key systems enriched in the downregulated genes were axon guidance, regulation of synapse structure or activity, and metanephric nephron development.

### Network-based systems biology analysis: protein−protein interaction analysis and functional enrichment

The 1480 DEGs with human orthologs are biologically related, forming a module within the network that comprises 1433 nodes and 7637 edges. This is a significantly higher number of edges than one that would be found by chance with an equivalent number of proteins (*P*<1×10^−16^), indicating a higher number of observed versus expected protein (or gene) interactions for a network of this size; thus, suggesting these genes include highly connected modules. Therefore, we used network clustering analysis to gain a more nuanced understanding of the biologic systems influenced by DEGs. This was performed by using the following steps, as described previously by us (Davidson et al., 2025): 1) creation of an organ-wide interaction network using the DEGs (both up- and downregulated) in the ECPR group, 2) clustering of the network to identify closely linked groups of DEGs, 3) gene set enrichment analysis on each cluster (Figs S1–S19). These clusters demonstrate differential expression of genes from a wide range of biologic systems in ECPR-exposed animals, including response to oxidative/nitrosative stress (Fig. 6A, Fig. S2), alteration of water and solute channels (Fig. 6B, Fig. S9), and renal angiogenesis (Fig. 6C, Fig. S13). We appreciated additional altered regulation of clusters of proteins involved in stress/starvation/circadian response (Figs S3, S7), hemostasis/coagulation (Fig. S5), protein folding (Fig. S6), cytokine/chemokine response (Fig. S11), carbohydrate and lipid metabolism (Figs S1, S8), ribosome biogenesis (Fig. S14), neuron development and signaling (Figs S15, S16), and microtubule organization (Figs S17, S19).

**Fig. 6.**
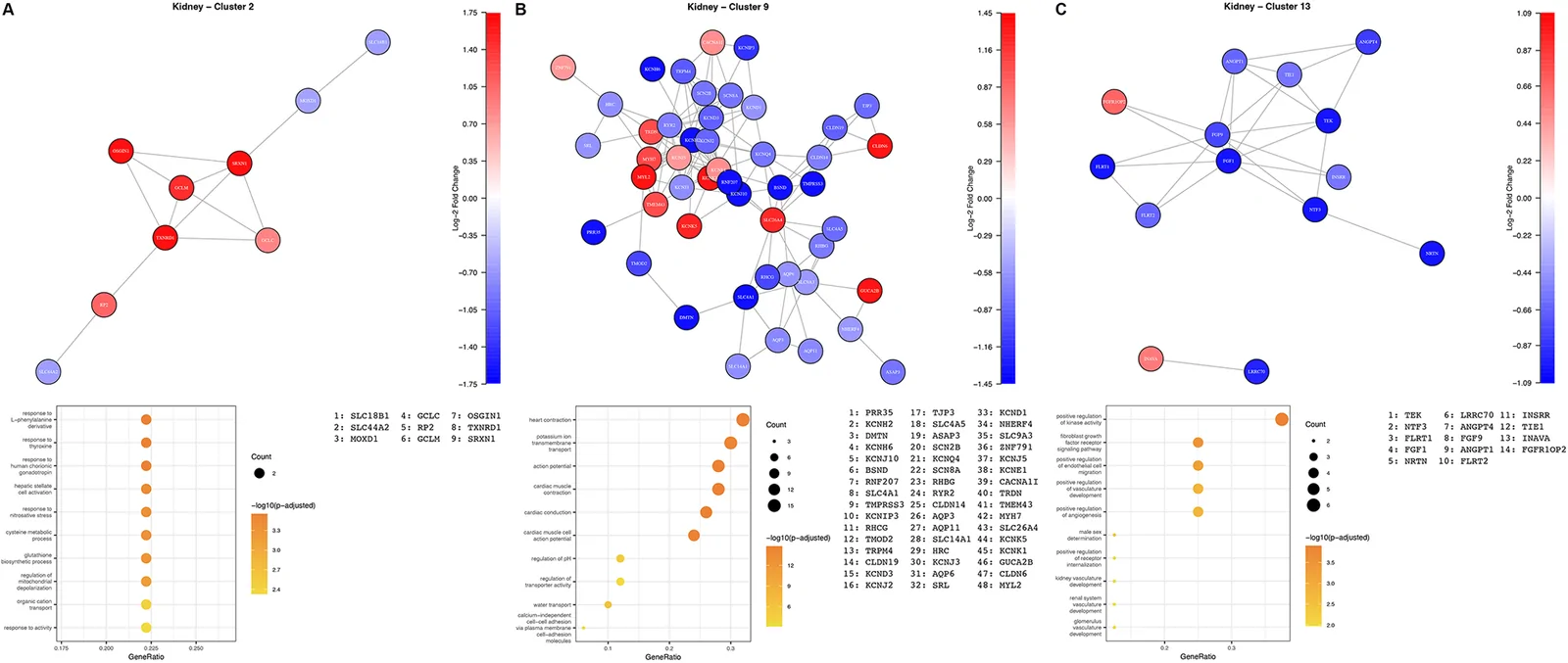
**Network connections and enrichment of select clusters.** (A) Cluster of up- and downregulated differentially expressed genes (DEGs) regulating oxidative and nitrosative stress with corresponding biologic system enrichment. (B) Cluster of up- and downregulated DEGs regulating renal angiogenesis with corresponding biologic system enrichment. (C) Cluster of up- and downregulated DEGs regulating water and solute channels with corresponding biologic system enrichment.

## DISCUSSION

Here, we reported a pediatric model of AKI following ECMO that results in histologic injury, biomarker (NGAL, serum creatinine) and transcriptomic disruption, with early evidence of physiologic injury, and which may be used to study the mechanisms underlying this key ECMO morbidity. Estimates in the literature of AKI from all-indication ECMO are as high as 70%, (Foti et al., 2021; Thongprayoon et al., 2019), and even higher for ECMO in pediatric patients and for cardiac indications (74%) (Smith et al., 2009).

### Why pediatric ECMO

Cardiopulmonary support with ECMO and ECPR specifically is a common, and growing intervention. ECMO imports critical changes to physiology, including loss of arterial pulse pressure, regional hypoperfusion and reperfusion injury. When coupled with the systemic inflammatory response by exposure to exogenous material, circuit hemolysis, and altered clotting and bleeding, ECMO alone results in a multiorgan systemic response that can lead to organ dysfunction and cellular stress. Studying these effects in humans is challenging, as investigation is limited to circulating and other peripheral markers. Animal models of ECMO are important for the comparison of circulating markers with tissue-level investigation through tissue harvest, and investigators have developed animal ECMO models in response to this (Ijuin et al., 2023). However, despite the fact that there are disproportionately more pediatric ECMO runs relative to the total population age distribution (ECLS International Summary of Statistics, accessed 17 October, 2023), there are relatively few ECMO or cardiopulmonary bypass models focusing on juvenile animals (Wittnich et al., 2001; Dhari et al., 2021; Ball et al., 2023). Pediatric patients have unique anatomy (e.g. the presence of thymic tissue) and physiology (e.g. higher cardiac output and O_2_ consumption per kilogram) in comparison to their adult counterparts. These age-specific changes likely extend to the transcriptome in ways not yet described or understood. Further, the effects of ECMO on pediatric patients may be lifelong, having a disproportionate burden on long-term health and the healthcare system. We chose juvenile pigs (∼10 kg) to address the unique age-specific responses to systemic physiologic stress for extracorporeal support.

### Why a pig animal model

Other research groups have developed ECMO models using various species, cannulation strategies and circuit types (Ball et al., 2023; Said et al., 2017; Conway et al., 2020; Gerfer et al., 2023). Our model utilizes pigs because of their favorable size, genetic profiles and, importantly, similar cardiovascular and renal anatomy, and comparable physiology to humans.

Apart from non-human primates, pig cardiovascular anatomy is most similar to humans and, for ethical and financial constraints, pigs are generally accepted as the ideal animal research subject in our field (Ijuin et al., 2023; Lelovas et al., 2014). They have a four-chambered heart with similar in- and out-flows, differing most notably in the coronary anatomy and position of the heart within the chest, most likely because of the shape of the thorax and quadruped stance. They generate similar hemodynamics to a size-matched human, with comparable heart rate, blood pressure and cardiac output (Lelovas et al., 2014).

Important for our study, pigs have also similar renal anatomy and physiology compared with humans. Pig and human kidneys comprise the same basic structures, including multilobular kidneys with nephrons made up of renal tubules and collecting ducts (Giraud et al., 2011). Hower, some slight differences between pig and human kidney exist: First, since the pig kidney has fewer total nephrons and fewer long-looped nephrons, pigs excrete more urine per kilogram than humans. Second, pigs have, therefore, a lower glomerular filtration rate when indexed to body size, (Kaissling and Kriz, 2010), resulting in a wider range of normal serum creatinine levels, i.e. between 0.6 and 2.7 mg/dl in our literature review (Hannon et al., 1990; Ekser et al., 2012). Nevertheless, pigs are generally accepted to be remarkably similar to humans, emphasized by the recent use of pig to human xenotransplants (Chase, 2024).

Given the transcriptomic focus of this study, we also sought a sufficiently similar genetic background. Pigs and humans share vital genetic similarities, and some genetic mutations in pigs and humans result in similar pathologic phenotypes, making them an ideal model for studying mechanisms of injury at the gene and RNA levels (Chade et al., 2018; Groenen et al., 2012).

### Kidney injury titration and model validation

In clinical scenarios, patients are often cannulated to ECMO in ECPR or in peri-arrest situations with variable periods of warm ischemic time based on time to recognition of clinical deterioration and institutional variation in time to ECMO cannulation. Our goal was to titrate a warm ischemic time that would induce clinical evidence of organ injury without being hemodynamically devastating to best mimic the ischemic stress experienced by patients. To identify the ideal arrest period, our model included variable periods of warm ischemia ranging from 5 to 20 min, followed by a set 4-h ECMO. Anesthetic agents alone have been associated with AKI, often secondary to lowered mean arterial blood pressure and, therefore, renal perfusion pressure (RPP) (Lichter et al., 2024). Our mechanical ventilation controls experienced inhaled isoflurane and had an average mean arterial pressure of 56±12 mmHg, which could lead to lower RPP. Despite this, there was no blood biomarker evidence of model-induced AKI in any control animals; in fact, control animals with a baseline elevation in urine NGAL secondary to pre-procedural fasting showed decreasing urine NGAL levels over the course of experimental observation. Histologic kidney injury was quite minimal in control animals. Of the 20% of samples with any degree of injury, four of the six animals had grade 1 injury without vacuolization, and two animals had grade 2 injury alone and only in 1 of 3 slices, suggesting that the anesthesia and mechanical ventilation aspects of our model only contributed minimally to any observed kidney injury.

We then progressed to variable periods of arrest. The 5-min arrest period induced appreciable kidney injury as detected by increased levels of NGAL alone, while other clinical metrics failed, with no increase in proteinuria or creatinine and normal urine output for size. The early rise of urine NGAL, a more modern clinical marker for AKI, highlights the benefits of ongoing investigation and validation of novel biomarkers for early detection of AKI. The only change between pre- and post-ECMO samples on urinalysis was a trace increase in hematuria, which would clinically be unlikely to warrant significant concern for AKI. By histology, kidney sections from this animal had some tubular dilatation and epithelial flattening, underscoring the degree to which sub-clinical organ injury may accumulate unrecognized in patients when only evaluating more traditional biomarkers. In contrast, pigs with longer ischemic times had increasing evidence of histologic injury that correlated with length of arrest time. An arrest duration of 10 min resulted in an intermediate histologic phenotype with increased tubular dilation/epithelial thinning and the appearance of epithelial blebs. While urine NGAL levels did rise in this piglet, they did not rise as early and as substantially as in the others, only reaching the greater-than-maximal detection level by the pre-euthanasia time point. Arrest times of 15 and 20 min were marked by higher degrees of injury when using microscopy, with all tissue samples demonstrating some tubular dilatation and/or epithelial flattening and most samples displaying ≥50% injured tubules. Overall, arrest times of 15 to 20 min were sufficient to induce histologic evidence of kidney injury, with early and substantial changes in urine NGAL, and early evidence of other clinical organ injury – an ideal threshold for future AKI biomarker investigation.

### Comparing the global transcriptome of ECMO and control kidneys

The kidney transcriptome in ECMO-supported pigs is distinct from mechanically ventilated controls, indicating a significant and early response at RNA level in the kidney following cardiac arrest and ECPR. As a model development study, we sought to identify the optimal arrest time for induction of kidney injury. Any period of cardiac arrest followed by 4 h of ECMO support resulted in differential transcriptome expression compared to controls. Pigs with shorter arrest times of 5 and 10 min showed intermediate separation from controls, while pigs with a 15- and 20-min arrest period demonstrated further separation from controls, and similar transcriptomic phenotypes when compared with each other. These same pigs demonstrated more significant and consistent cross-sample histologic evidence of AKI with widespread tubular dilatation and epithelial flattening, and frequent bleb formation. The transcriptomic fingerprint correlated with histologic kidney injury but was less closely aligned with biomarkers or changes in urinalysis, further emphasizing the shortcomings of following creatinine and other traditional biomarkers, whose dysregulation is often delayed as they rely on decreasing glomerular filtration. In contrast, transcriptome changes in our model occurred within 4 h of cardiac arrest and ECPR-induced injury and, thus, are ready for novel biomarker exploration.

### Systems biology disruption in ECMO-AKI

Initial analysis of the systems biology of DEGs demonstrates widespread alterations in gene expression across biologic systems, including metabolism, coagulation and hemostasis, chemokine and cytokine signaling, and cellular stress responses at multiple points (oxidative stress, starvation response). RNA coding for proteins with organ-specific functions, such as renal angiogenesis and neuronal development and signaling, were also enriched. We also appreciated the enrichment of altered responses at structural levels, including changes to protein folding, cell junction assembly and microtubule organization. These global transcriptomic shifts are consistent with the existing literature in AKI of diverse etiologies, which describes the upregulation of inflammatory and profibrotic pathways, (Wen et al., 2023), changes in fatty acid metabolism, and regulation of solute and anion channels (Melo Ferreira et al., 2021) and oxidative stress and hypoxia interferon response (Hinze et al., 2022). Overall, the similar global transcriptome patterns experienced by our ECMO pigs to other animal models are reassuring of the model efficacy.

Beyond global transcriptomic shifts, our model also identifies both previously studied as well as novel genes associated with AKI. Among the most-differentially expressed genes that are significantly different from controls are genes with known association in AKI and genes involved in ischemic stress response, i.e. *HMOX1* (Zager et al., 2012), *FOSL1* and *FOSB* (Cuarental et al., 2023) and *BACH1* (Yamaoka et al., 2017). We also identified genes involved in global patterns of immune response, such as chemotaxis, cell adhesion and leukocyte activation, which have been identified by Tang et al., by performing single-cell RNAseq in an ischemia-reperfusion-injury model of AKI (Tang et al., 2023). That group also identified several unique pro-apoptotic genes (*USP47*, *RASSF4*, *EBAG9*, *IER3*, *SASH1*, *SEPTIN7* and *NUB1*) that are upregulated. Our enrichment analysis only identified two directly pro-apoptotic genes and did not confirm those identified by Tang and colleagues, possibly owing to the comparative severity in AKI, as the study by Tang et al. included patients histologically showing frank necrosis and because apoptosis might, therefore, represent more-advanced or longer-lasting injury. Our ECMO-AKI pigs were enriched in several novel AKI-associated genes based on our literature review. Those most highly enriched and most significantly different from controls (*OSER1*, *RPS6KA2*, *ARRDC4*, *ELL2*, *KCNH2*, *WSCD1*) govern a variety of cellular processes, including oxidative stress response, receptor endocytosis and K^+^-transport via ion channels. Although there may be some confounding of potassium transporter regulation in our model, which included exogenous K^+^ administration to induce arrest, these genes of interest warrant further investigation to understand their role in AKI and to assess if their expression is unique to the kidney.

### Limitations

This study was designed for model development with different interventions (i.e. cardiac arrest length) for each animal and is, therefore, not powered to make definitive statements on the degree of kidney injury or RNA response for any individual arrest time. Future studies investigating multiple animals and only one length of cardiac arrest (15 or 20 min) are needed to better characterize the transcriptome in ECMO-associated AKI. Additionally, although the technique of cannulation prior to arrest was chosen to limit variability in time to cannulation, this does not mimic real-world ECPR situations, in which healthcare providers attempt chest compressions through the surgical cannulation process. The latter provides some degree of circulation and O_2_ delivery, although the time until ECMO cannulation is often longer than our arrest period, which may mitigate some of these differences. Finally, although we believe tissue-level omics is an overall strength and indication for an animal model, particularly for the study of injury mechanism and therapeutic development, organ-level biomarkers alone lack diagnostic clinical translatability. Additional studies are needed to correlate these gene expression changes with downstream protein shifts and to correlate tissue-level biomarkers with circulating protein biomarkers for future use at the bedside.

## CONCLUSIONS

AKI following ECMO support in pediatric patients is clinically significant to acute mortality and associated with lifelong morbidity. We developed a pediatric ECMO−ECPR model to induce clinical, histologic, biomarker and transcriptomic kidney injury. Through RNAseq of kidney tissue, we identified almost 2000 DEGs across biologic systems, representing a wealth of novel targets for mechanistic exploration and therapeutic development. Although the model presented here focuses on AKI, this study also serves as a proof-of-concept of our model design and inducible organ injury, and is easily adaptable to other organ systems.

## MATERIALS AND METHODS

The present protocol was approved by the Institutional Animal Care and Use Committee at the University of Colorado. Experienced practitioners, including certified veterinarians, provided supervision and assistance in animal care before and during the experimental procedures. Thirteen pediatric pigs (*Sus scrofa domestica*), weighing 8-11 kg were used for the study, with a near-equal balance of male and female pigs (*n*=6 and *n*=7, respectively), aged 5–7 weeks. Of those 13 pigs, four were supported on ECMO and nine pigs, shared with another study cohort, were mechanical ventilation-only controls.

### ECMO circuit configuration

We designed an ECMO circuit with components similar to those used in the pediatric cardiac ICU at Children's Hospital Colorado. Briefly, the venous limb drained deoxygenated blood via a 12 French venous cannula with 6.35 mm (1/4 inch) sterile polyvinyl tubing followed by a step-up connector to sterile 9.53 mm (3/8 inch) polyvinyl tubing (all Medtronic, Minneapolis, MN, USA) and a multi-way stopcock for three purposes: 1) continuous venous inlet pressure monitoring 2) connection to a ‘bridge’ between the venous and arterial limbs and 3) for continuation of the venous limb to a Maquet Rotaflow centrifugal pump (Getinge, Göteborg, Sweden) and then to the Sorin EOS membrane oxygenator (LivaNova, London, UK) with Cincinnati Sub-Zero ECMO 333W Heater for temperature regulation (CSZ, Cincinnati, OH, USA). Upon exit from the oxygenator, oxygenated blood passed through a bubble detector and flow probe (Transonic Systems Inc., Ithaca, NY, USA) before reaching the animal via a 10F arterial cannula using a 3/8″ to 1/4″ step-down connector. Additional connections to the system included a three-way stopcock connected to a 1-l crystalloid and blood priming bag (Baxter, Deerfield, IL, USA) and a stopcock for additional transfusion in the setting of active ECMO flow.

### Surgical preparation

Pigs were pre-medicated with intramuscular ketamine (30−35 mg/kg), acepromazine (1.1−2.2 mg/kg), and atropine (0.05 mg/kg) (VetOne, Boise, ID, USA) before anesthesia via mask ventilation with 5% isoflurane in 100% O_2_ at a constant flow rate of 1 l/min. Pigs were then intubated and maintained with 1.5−2% isoflurane in 89−99% O_2_ prior to ECMO initiation. During ECMO support, we transitioned pigs to propofol (10 mg/ml) (Northstar Rx, Memphis, TN, USA) and fentanyl (50 μg/ml) (West-Ward Pharmaceuticals, Eatontown, NJ, USA). We completed surgical dissection and cut into the right groin for placement of femoral venous and arterial catheters for medication administration, laboratory sampling and continuous blood pressure monitoring. We placed a foley for continuous urine output monitoring directly into the bladder via puncture and secured with a purse-string suture. Simultaneously, we dissected the right lateral neck for ECMO cannulation using surgical cauterization and blunt dissection until the external jugular and internal carotid were free of surrounding adventitia. We ensnared the free vessels proximally and distally, and applied tension to cease blood flow. We first directly opened the internal carotid artery with a scalpel, opening ∼50% of the vessel diameter. We then placed a 10F arterial cannula into the vessel, tying the suture down onto the cannula for secure placement. We repeated the process in the external jugular vein, placing a 12F venous cannula such that the pig was fully cannulated to ECMO but with proximal clamps in place and without flow initiation.

### Cardiac arrest and ECMO support

Animals were treated with 500 IU/kg of intravenous heparin (10,000 U/ml; Northstar Rx), with additional doses administered as needed to achieve a goal activated clotting time of >300 s. Upon successful cannula placement, we connected pigs to the ECMO circuit without initiating flow, allowing for tight control of total ischemic time. We then induced cardiac arrest through an intravenous bolus of 3–8 mEq/kg of potassium chloride (KCl), stopping the bolus upon induction of ventricular fibrillation or asystole. We left the pigs in ventricular fibrillation or asystole for an increasing period of warm ischemic time (5, 10, 15 or 20 min, with one pig per condition), titrating the arrest time to induce goal organ injury. Following completion of the warm ischemic period, we initiated ECMO flow at 100−110 ml/kg/min by slowly increasing the centrifugal pump until reaching a target flow of 100−130 ml/kg/min. A sweep gas of 100% O_2_ flowed at 1 liter per minute (LPM). At the time of ECMO initiation, vasoactive-inotropic support was initiated with epinephrine, milrinone and/or vasopressin (McKesson, Westminster, CO, USA), as appropriate based on hemodynamics and ECMO pressure/flow. Animals were maintained on ECMO at normothermia (37°C) for 4 h with titration of vasoactive medications to achieve a goal mean arterial pressure of 45−65 mmHg and goal central venous pressure (CVP) <10 mmHg, balancing renal perfusion pressure (RPP) against the risk of hemorrhage. From a ventilation standpoint, we monitored arterial blood gases using a pH strategy, targeting a goal pCO_2_ of 35−45 mmHg. Pigs were ventilated at ∼13 ml/kg and a positive end-expiratory pressure of 6 cm H_2_O to minimize atelectasis. ECPR pigs were ventilated for 7−8 h, with total ventilation time varying slightly due to differences in surgical cannulation and line placement times. Nine pigs were intubated and mechanically ventilated for 8 h, serving as controls. We sampled blood and urine pre-procedurally, hourly while on ECMO and just before euthanasia.

### Tissue harvesting and processing

For tissue harvest, we collected the right kidney under deep sedation immediately prior to euthanasia and then passively drained it of blood prior to processing and storage. A portion of the cortex was flash-frozen in liquid nitrogen and stored at −80°C until homogenized and used for total RNA isolation using the RNeasy Mini Kit (Qiagen, Santa Clarita, CA, USA) as described in the manufacturer's protocol. RNA concentrations were quantified at 260 nm, and the purity and integrity were determined using a NanoDrop (Thermo Fisher Scientific, Waltham, MA, USA). Samples underwent whole-transcriptome RNA sequencing (RNAseq) at the University of Colorado Genomics Shared Resource (Aurora, CO, USA). RNA purity, quantity and integrity were determined with NanoDrop and TapeStation 4200 (Agilent Technologies, Santa Clara, CA, USA) analysis prior to RNA-seq library preparation. The Universal Plus mRNA-Seq library preparation kit with NuQuant was used (Tecan, Morrisville, NC, USA) with an input of 200 ng of total RNA to generate RNA-Seq libraries. Paired-end sequencing reads of 150 bp were generated on NovaSeq 6000 sequencer (Illumina, San Diego, CA, USA) at a target depth of 80 million paired-end reads per sample. Raw sequencing reads were de-multiplexed using bcl2fastq.

A second, full-thickness slice of the kidney, including the cortex and medulla, was separately fixed overnight in 10% formalin, paraffin-embedded, and sectioned at 4 μm. Sections were then stained with hematoxylin and eosin (H&E) and three sections per animal were evaluated for histologic evidence of kidney injury by an observer unaware of the experimental conditions. Samples were evaluated for the severity of kidney injury by two observers unaware of the scorers by using previously published methodology (Murphy et al., 2009; Wang et al., 2015; Patel et al., 2011; Moledina et al., 2017; Davidson et al., 2019). At low magnification (2×), sections were evaluated for tubular dilatation and epithelial flattening, and given a grade between 0 and 4 based on the percentage of parenchyma affected, with 0=0−4.99%, 1=5–25%, 2=26–50%, 3=51–75% and 4=>75%.

Aliquots of femoral arterial blood were collected hourly for immediate point of care testing (POCT) of arterial blood gas and chemistry to assess the acid−base status, evaluate for electrolyte disturbances and to guide ventilator management and/or vasopressor titration. Additional aliquots were processed to collect serum, and then frozen and stored at −80°C. Urine was collected at matching time points and stored at −80°C until analyzed. Blood and urine were batch-analyzed upon completion of all experiments. Creatinine was measured using a commercially available DRI-CHEM analyzer (HESKA Lab System, Loveland, CO, USA). Urinalysis with sediment was performed on all collected urine samples at the Office of Laboratory Resources (OLAR) at the University of Colorado Medical Campus (Aurora, CO, USA). Additionally, urine NGAL levels were quantified by ELISA (Bioporto Diagnostics, Hellerup, Denmark) at three time points, i.e. at experimental start, midpoint and prior to euthanasia, with midpoint referring to 6 h elapsed time in controls and 2 h post arrest, which equates to 5–6 h elapsed time in ECMO animals, owing to variability in time for surgical preparation and cannulation). For the NGAL analysis, NGAL levels in some ECMO animals were above the detection limit (420 ng/ml) of the assay. In those cases, values were set to 420 ng/ml and the Mann–Whitney *U* test was used.

### Bulk RNAseq analysis

Methodology of RNAseq analysis has previously been described in depth by our group (Davidson et al., 2025) and is briefly summarized as follows.

#### Read alignment

The de-multiplexed fastq files were aligned using the Nextflow (Di Tommaso et al., 2017) based nf-core maseq pipeline (Ewels et al., 2020). Through this pipeline, Illumina adaptor sequences and low-quality sequencing bases were removed from the fastq files using the Trim Galore! implementation of Cutadapt. The resulting reads were aligned to the wild boar (Sus scrofa) genome using STAR (Dobin et al., 2013) and quantified using Salmon (Patro et al., 2017).

#### Differential expression analysis

A DESeq2 (Love et al., 2014) model utilizing IfcShrink with ashr (Stephens, 2017) was built to compare control samples to ECMO samples, as well as perform dimensionality reduction using PCA. A gene was considered significantly differentially expressed for log fold change >1.5, and Benjamini-Hochberg procedure (i.e. false discovery rate) corrected *P*-value of <0.05.

#### Network-based enrichment analysis

Due to the lack of sufficient gene annotations in swine, the sparsity of curated protein−protein interactions in swine networks and the end goal of determining ECMO effects in a human population, we performed the network clustering and enrichment analyses using human gene IDs. All Sus scrofa genes were converted to their Homo sapiens orthologs using Ensembl BioMart (Harrison et al., 2024). For each set of DEGs, we subset the STRING database (Szklarczyk et al., 2023) to those genes and performed clustering by using the Louvain algorithm implemented in the Python package scikit-network 0.32.1. Enrichment was performed on the genes contained in each cluster using the enrichGO function of the R package clusterProfiler (Yu et al., 2012).

## Supplementary Material

10.1242/dmm.052726_sup1 Supplementary information
