# Supplementary material for: Development of a pediatric pig model of extracorporeal cardiopulmonary resuscitation-induced acute kidney injury
Source: Dis Model Mech. 2026 Oct 2;19(9):dmm052726. doi: 10.1242/dmm.052726 (PMC13670352; doi:10.1242/dmm.052726)
Supplement: Supplementary file 1 [file dmm-19-052726-s1.pdf]

Figure – SM1

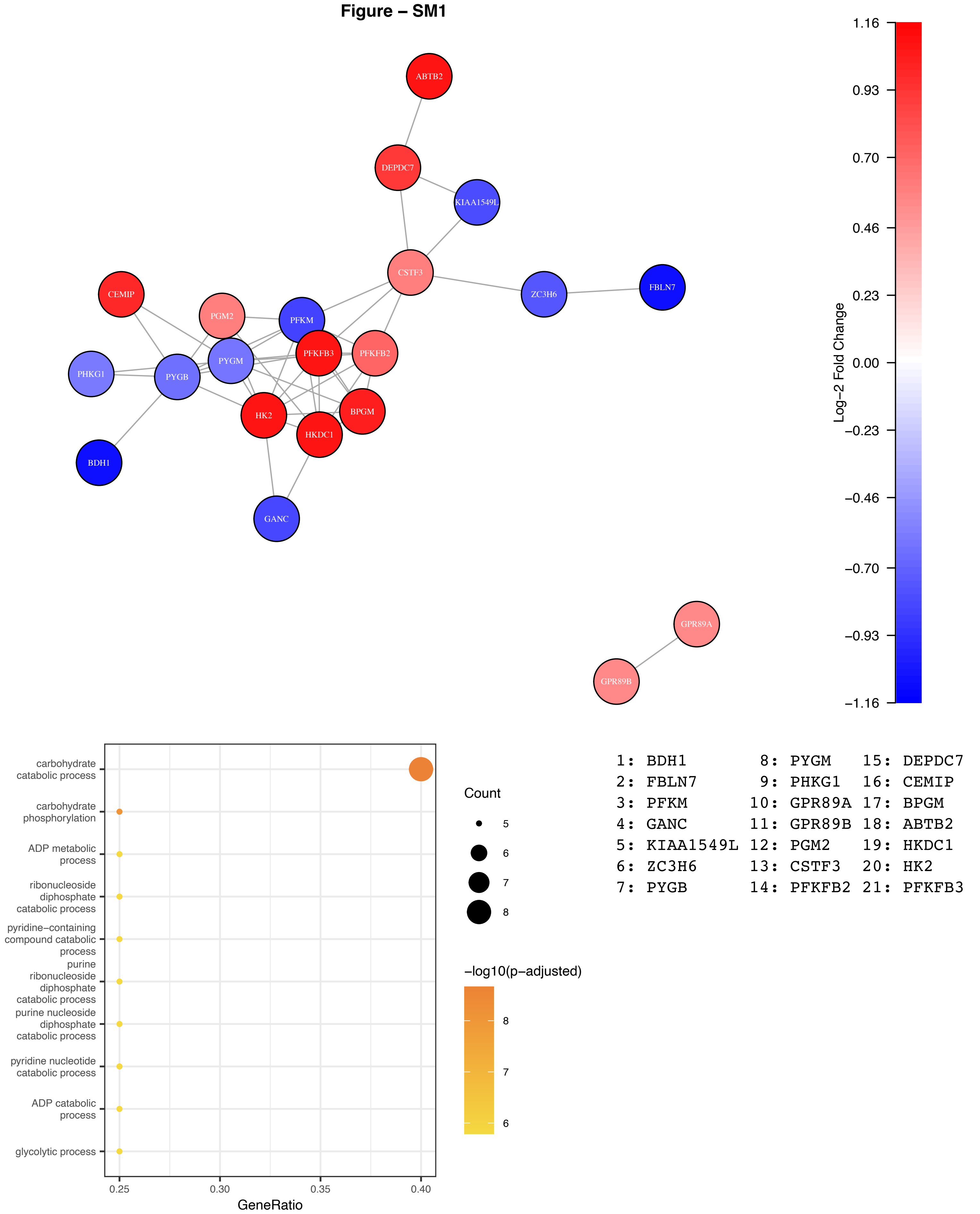

Fig. S1. DEG cluster of up- and downregulated genes regulating carbohydrate metabolism, glycogen turnover, and metabolic adaptation to fluctuating energy and oxygen availability.

Disease Models & Mechanisms • Supplementary information

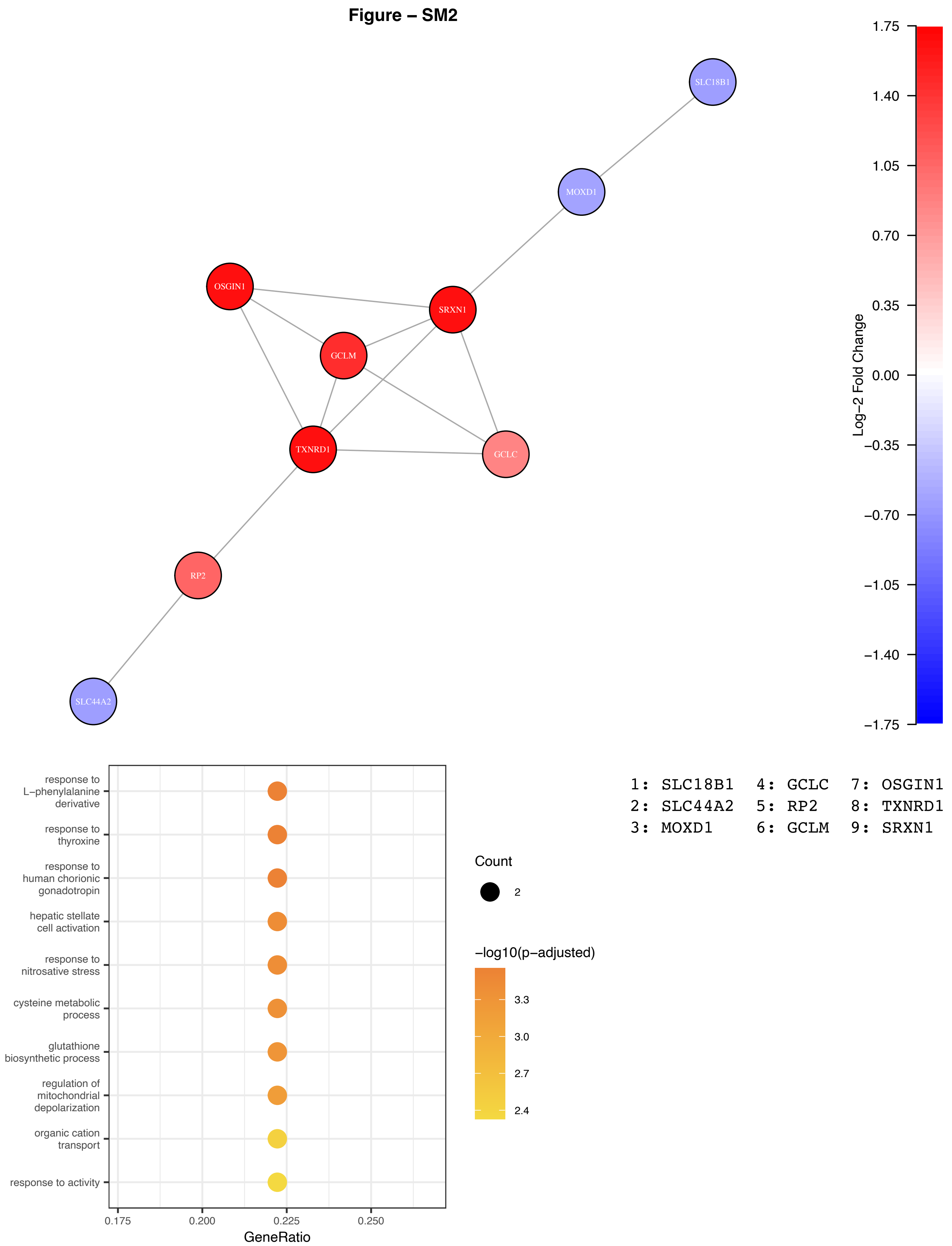

Fig. S2. DEG cluster of up and downregulated genes regulating cellular redox homeostasis, glutathione metabolism and oxidative stress defense.

Disease Models & Mechanisms • Supplementary information

Figure – SM3

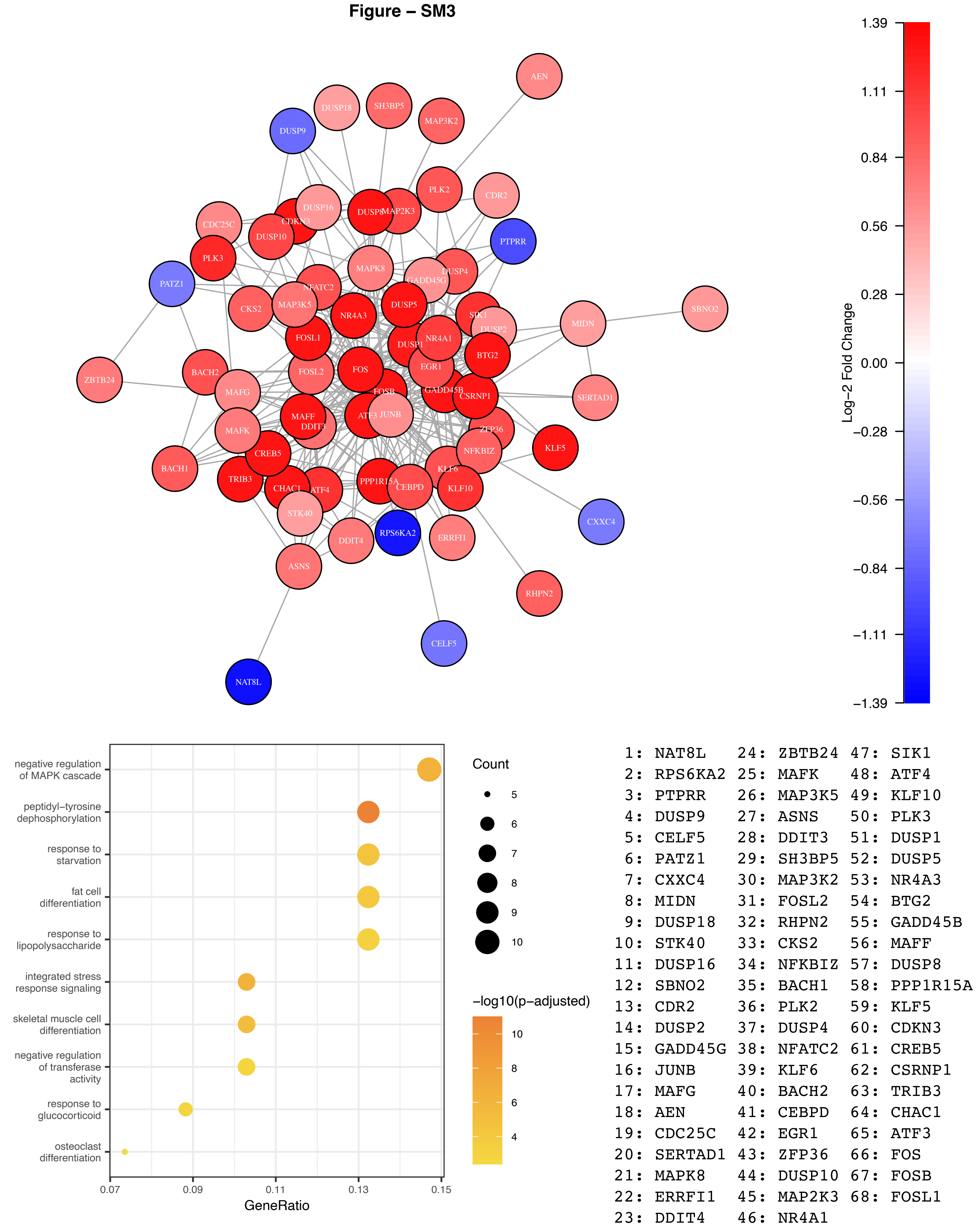

Fig. S3. DEG cluster of up- and downregulated genes regulating acute cellular stress response via MAPK signaling, integrated stress response, and DNA damage response, and cell checkpoint control.

Disease Models & Mechanisms • Supplementary information

Figure – SM4

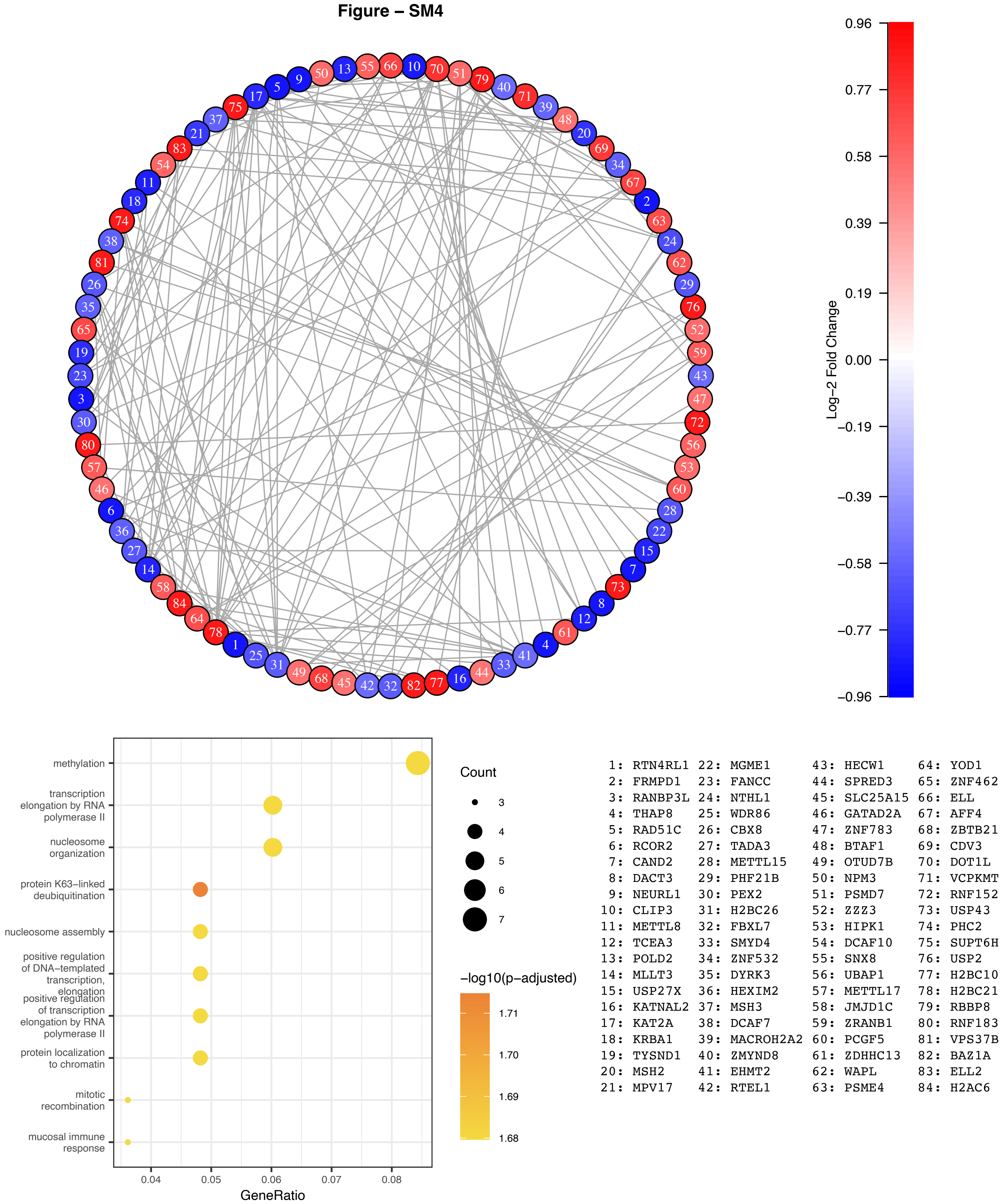

Fig. S4. DEG cluster of up- and downregulated genes regulating DNA repair, chromatin organization, transcriptional regulation, ubiquitin-mediated protein turnover and mitochondrial quality control.

Disease Models & Mechanisms • Supplementary information

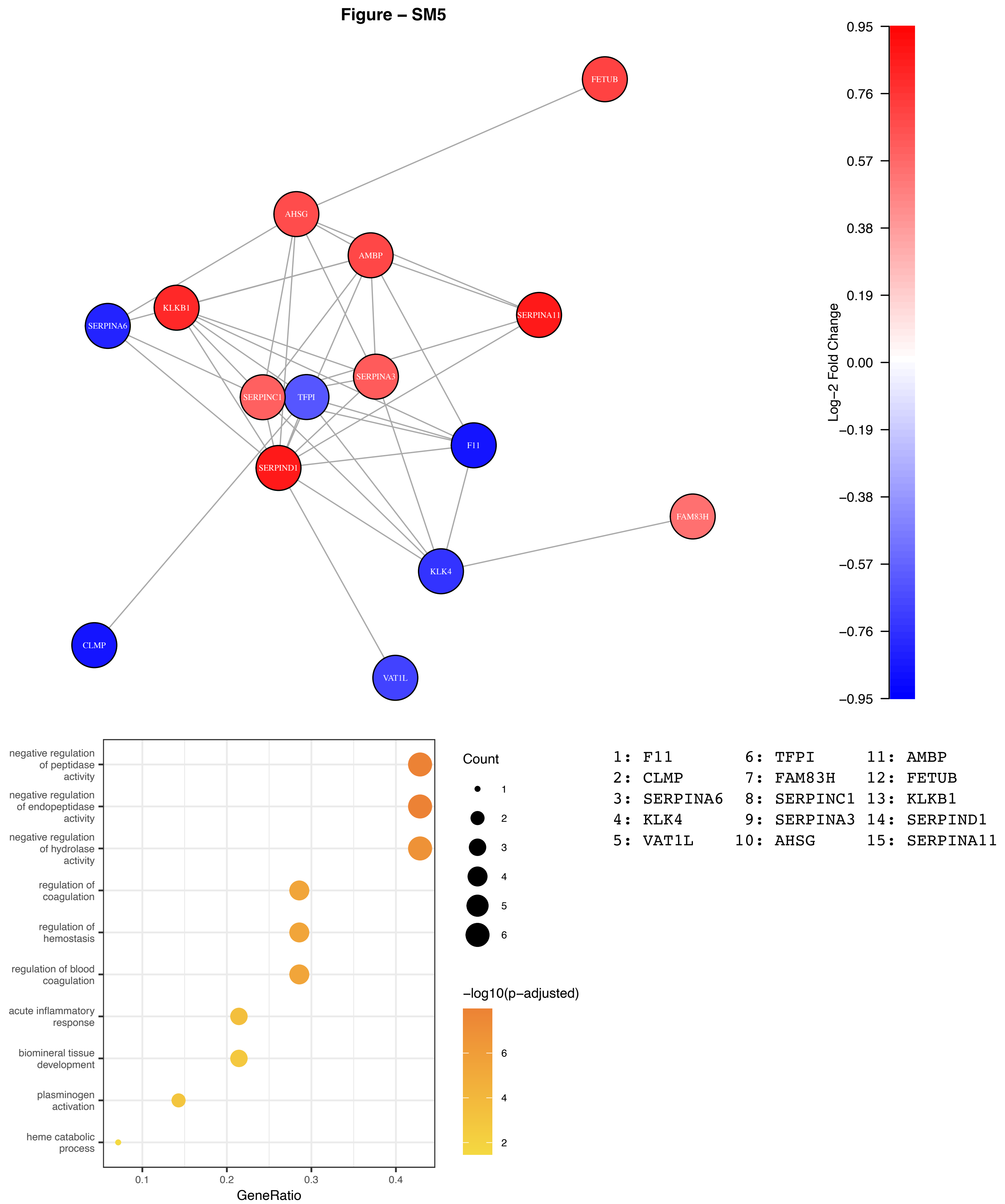

Fig. S5. DEG cluster of up- and downregulated genes regulating hemostasis via blood coagulation, protease inhibition and liver-derived plasma protein homeostasis.

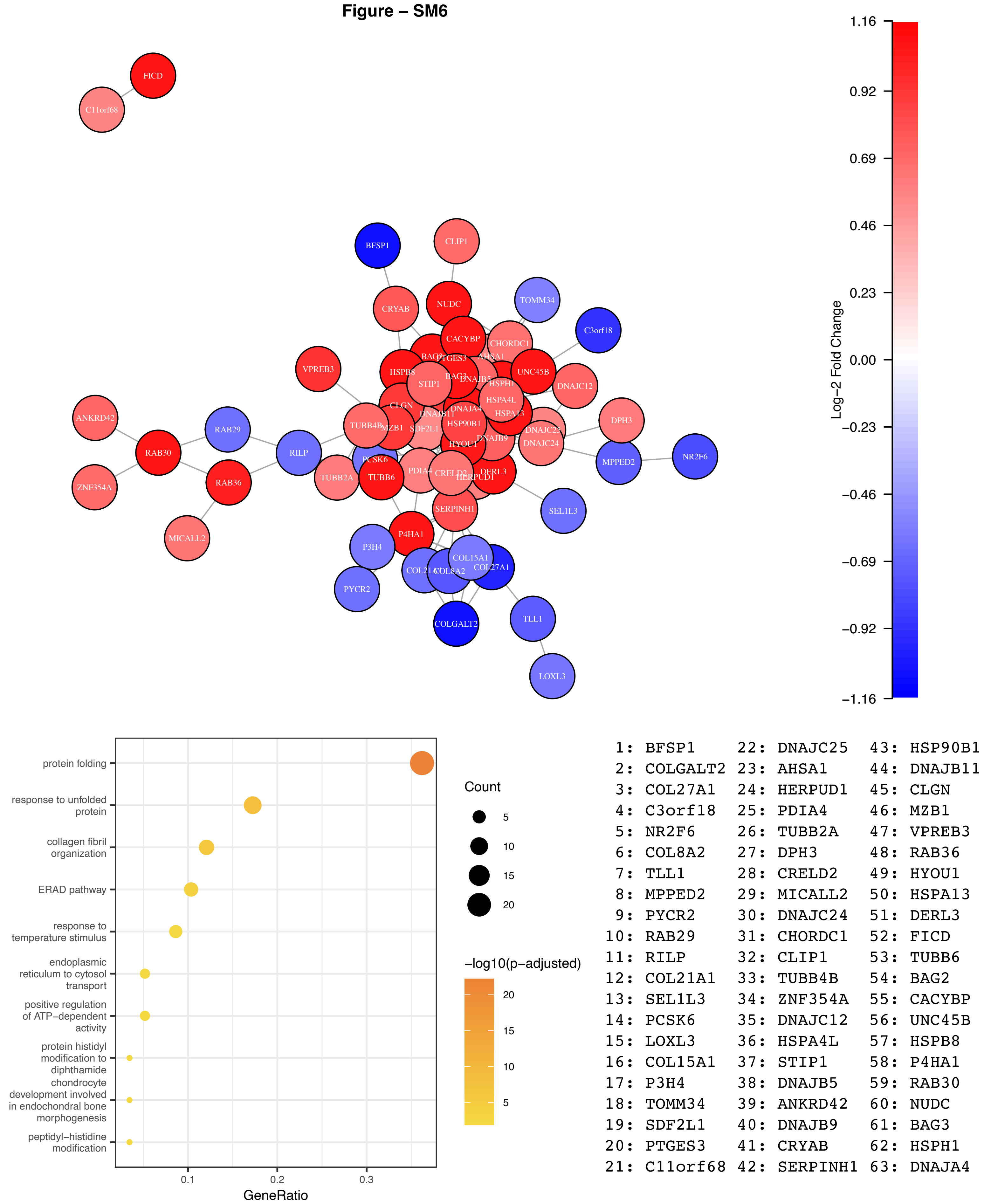

Fig. S6. DEG cluster of up- and downregulated genes regulating protein folding, endoplasmic reticulum (ER) proteostasis, collagen biosynthesis and extracellular matrix (ECM) assembly.

Disease Models & Mechanisms • Supplementary information

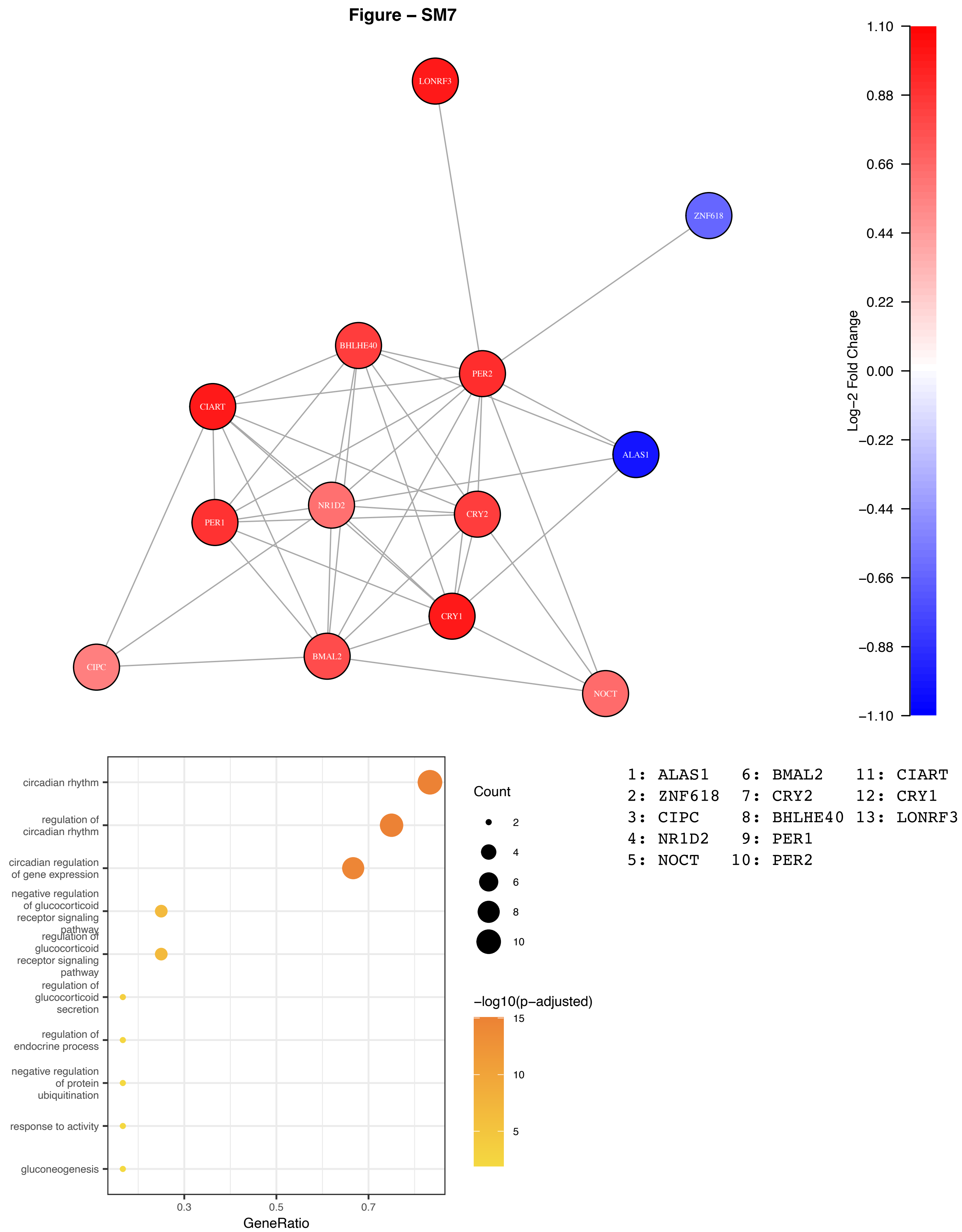

Fig. S7. DEG cluster of up- and downregulated genes regulating transcription–translation feedback loops for lipid metabolism, glucose homeostasis and circadian biology.

Disease Models & Mechanisms • Supplementary information

Figure – SM8

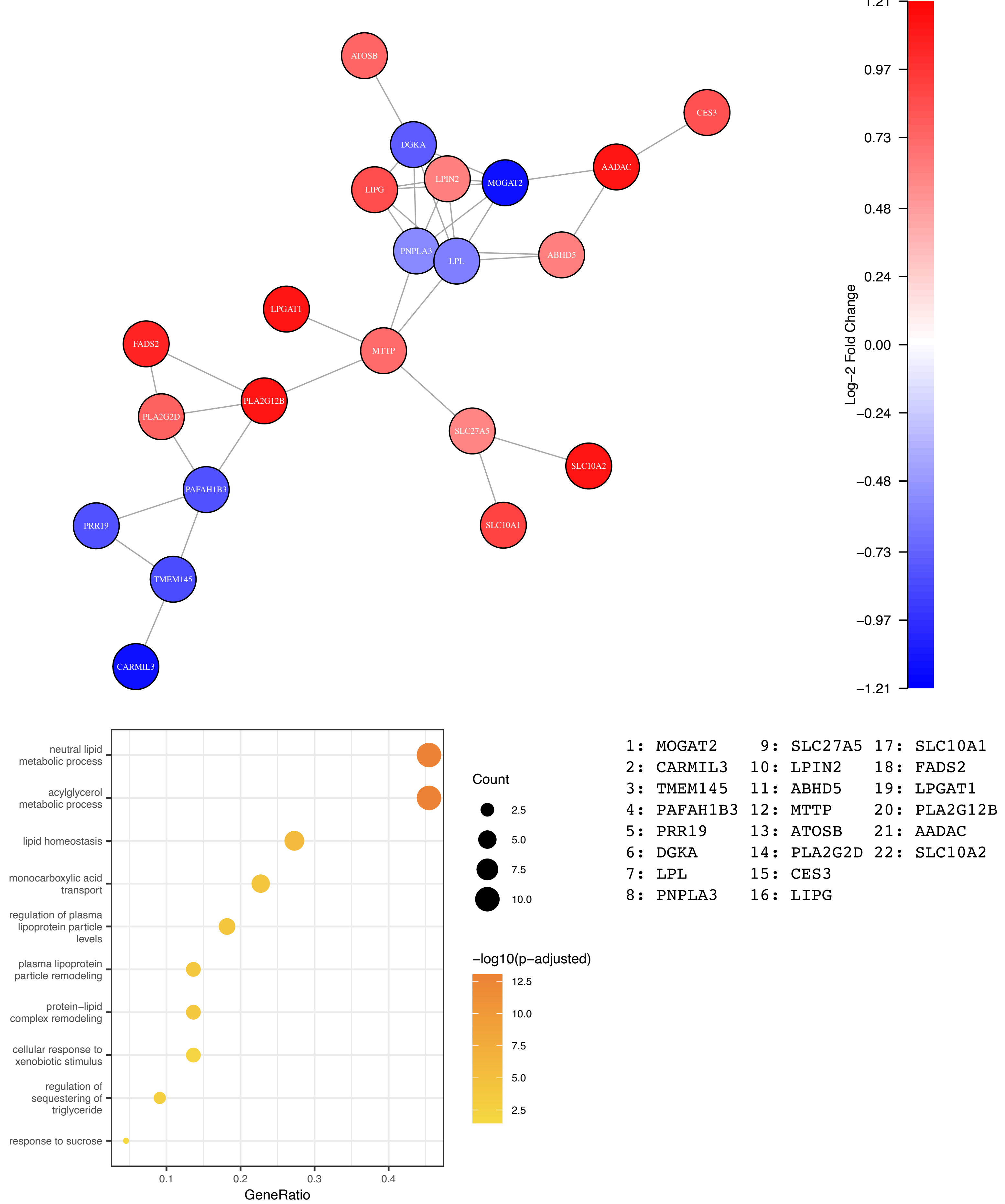

Fig. S8. DEG cluster of up- and downregulated genes regulating lipid absorption, triglyceride metabolism, phospholipid remodeling and lipid secretion.

Disease Models & Mechanisms • Supplementary information

Figure – SM9

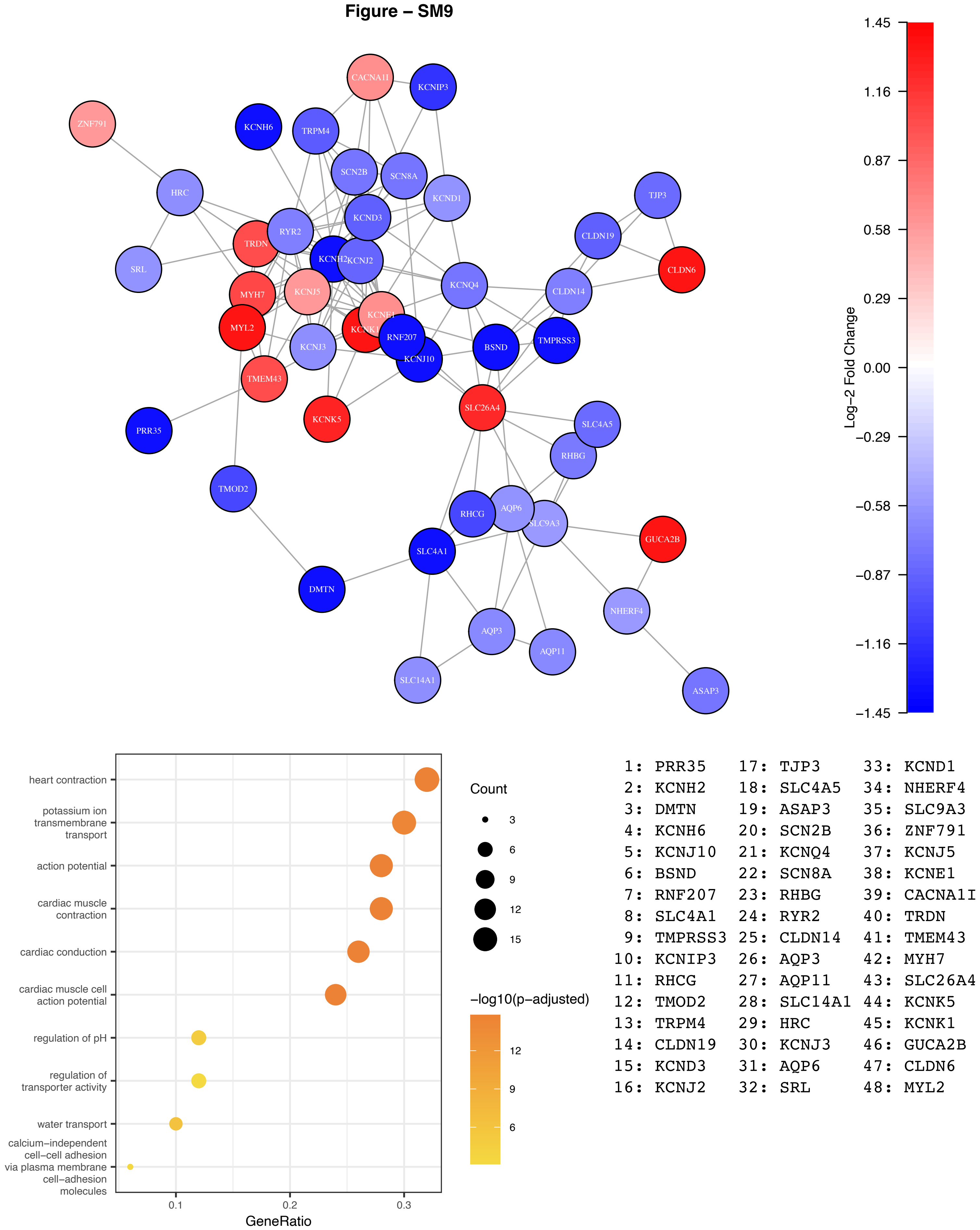

Fig. S9. DEG cluster of up- and downregulated genes regulating ion transport, epithelial electrolyte handling, membrane excitability maintenance of membrane potential and transepithelial ion homeostasis in the kidney.

Disease Models & Mechanisms • Supplementary information

Figure – SM10

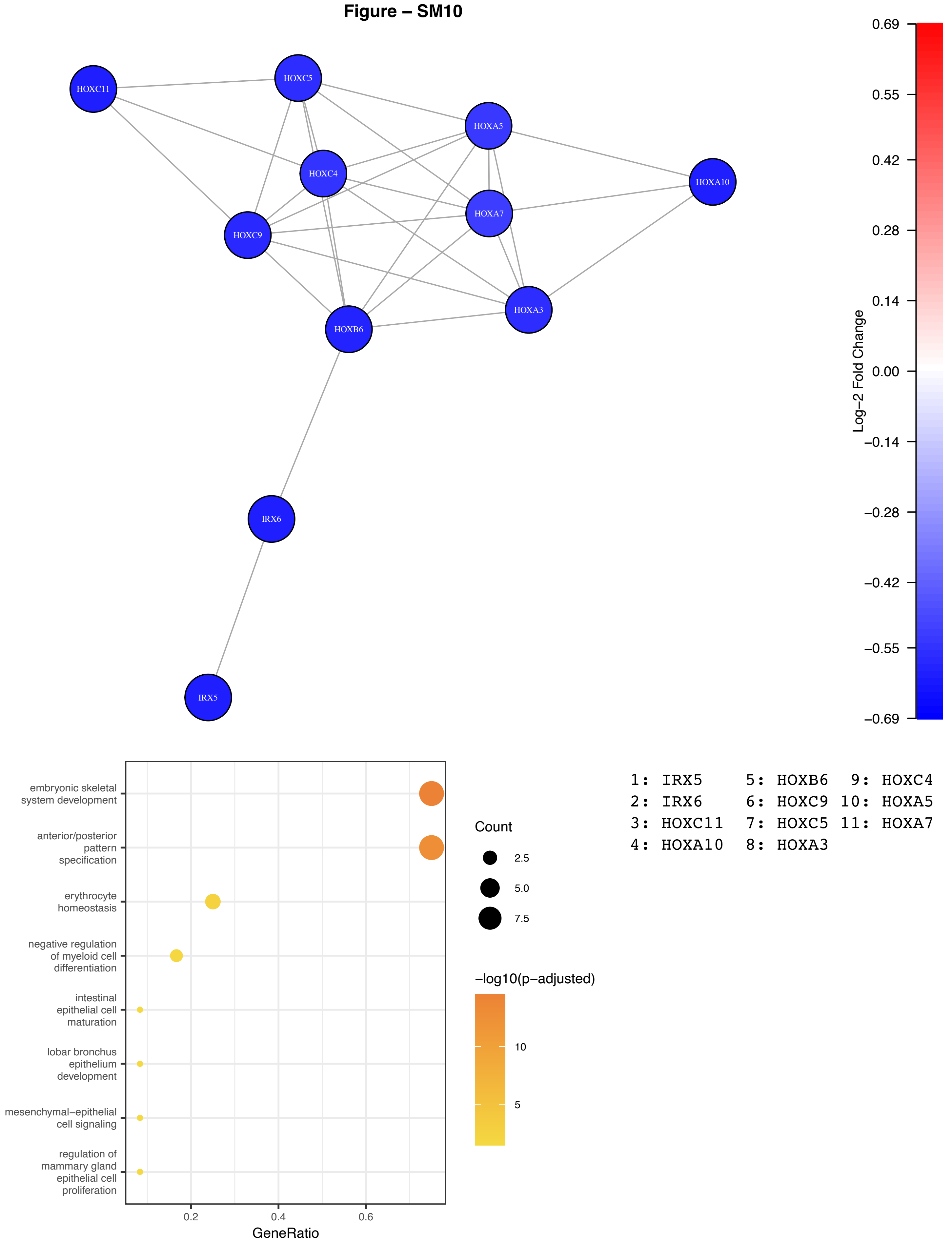

Fig. S10. DEG cluster of up- and downregulated genes regulating developmental patterning and cell fate specification module including embryonic body patterning, regional identity, morphogenesis, and differentiation.

Disease Models & Mechanisms • Supplementary information

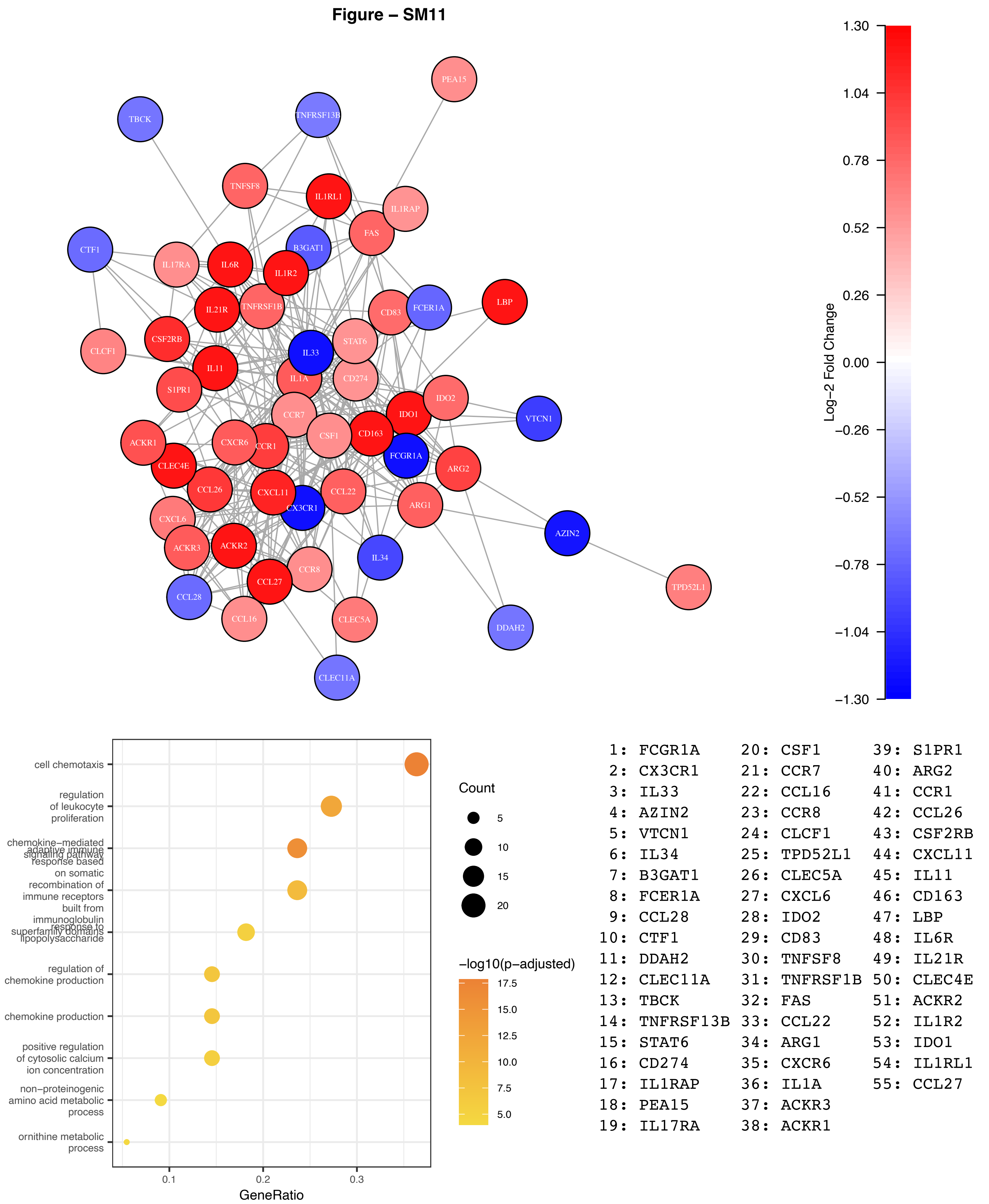

Fig. S11. DEG cluster of up- and downregulated genes regulating immune suppression and tolerance via chemokine signaling, cytokine responses and antigen presentation.

Disease Models & Mechanisms • Supplementary information

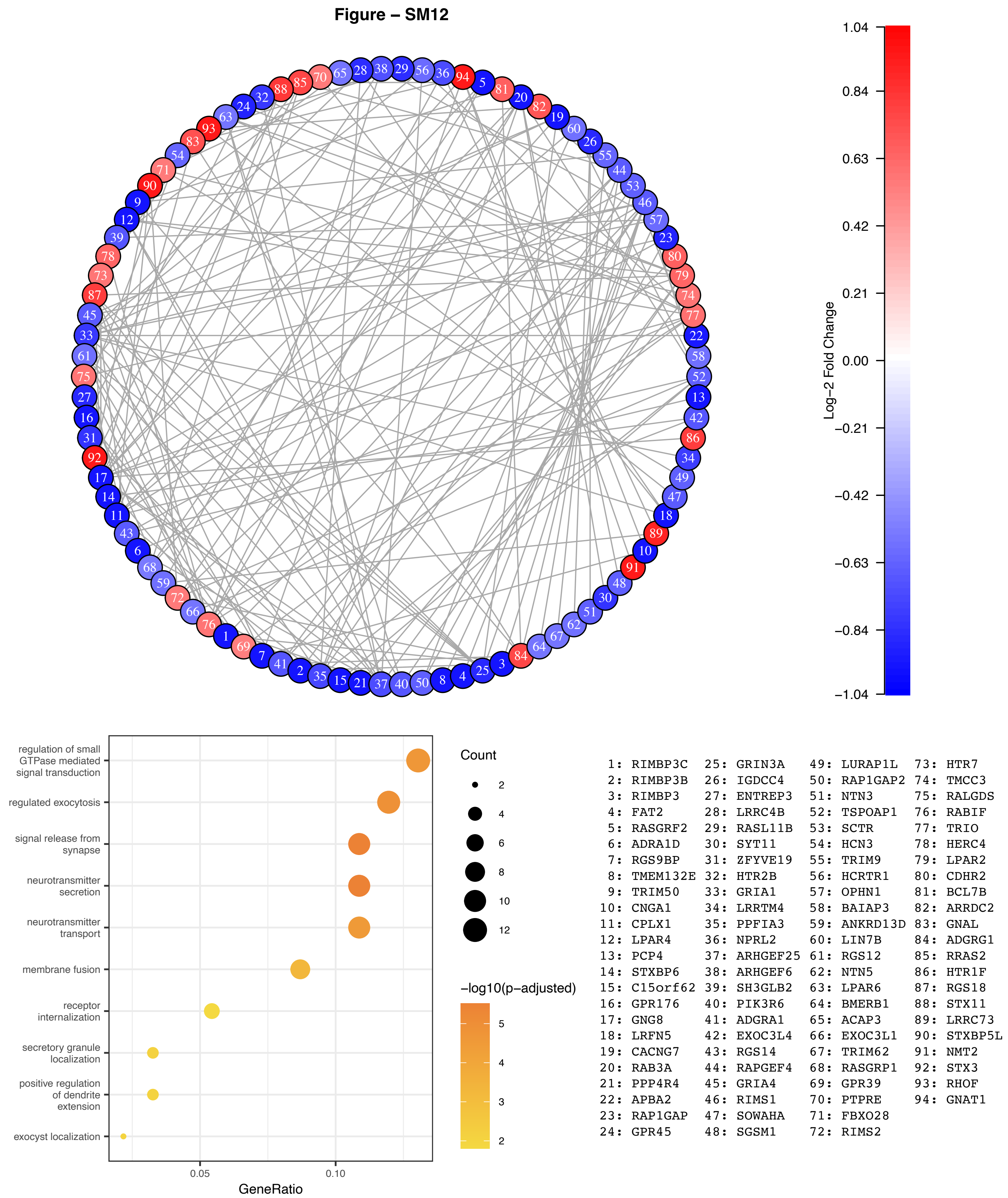

Fig. S12. DEG cluster of up- and downregulated genes regulating ion membrane potential and some specific synaptic vesicle release, neurotransmitter receptor signaling, G-protein coupled receptor (GPCR) pathways, and axon/dendrite development.

Disease Models & Mechanisms • Supplementary information

Figure – SM13

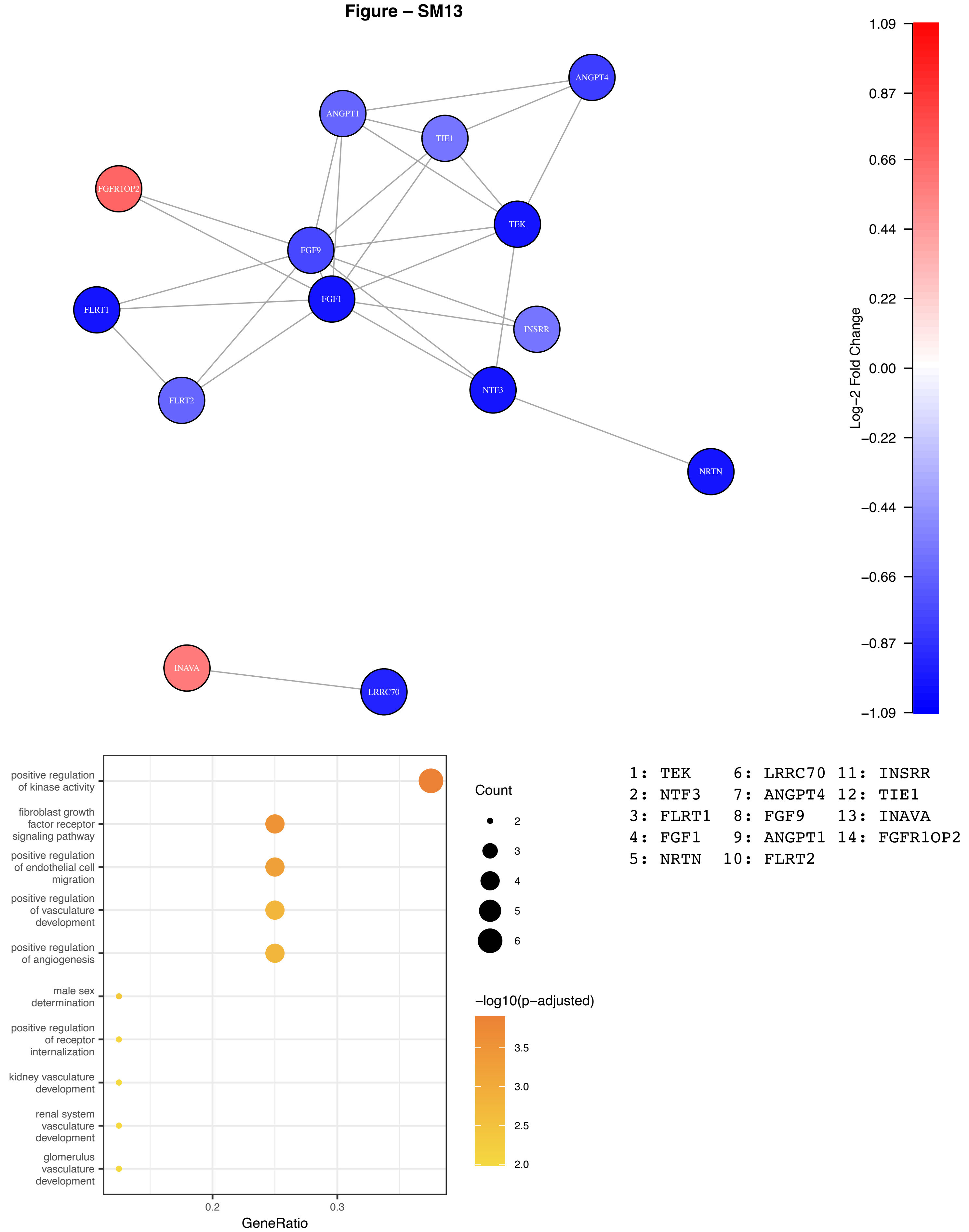

Fig. S13. DEG cluster of up- and downregulated genes regulating vascular development, angiogenesis and neurotrophic signaling module.

Disease Models & Mechanisms • Supplementary information

Figure – SM14

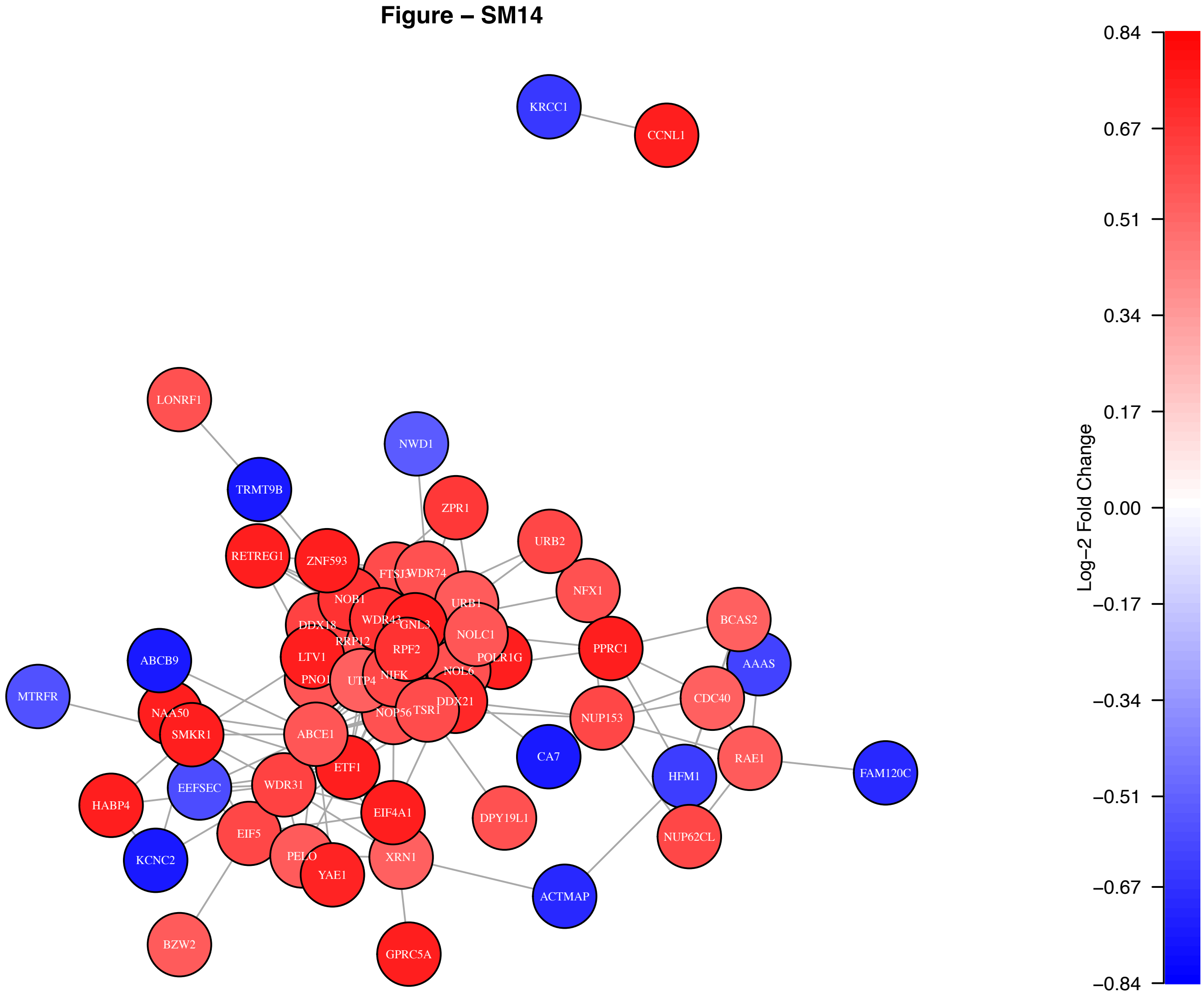

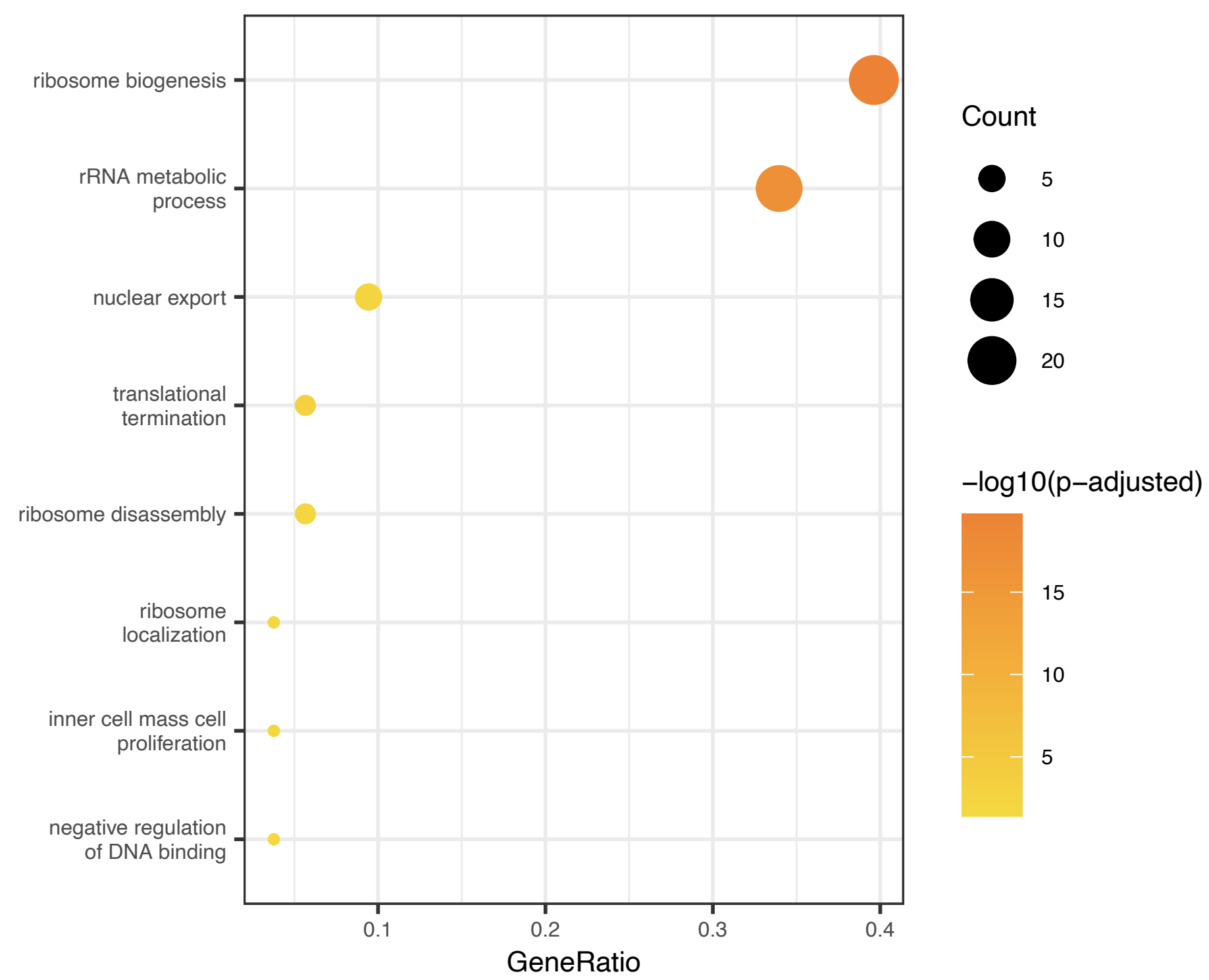

1: CA7	21: NOLC1	41: WDR43
2: TRMT9B	22: ABCE1	42: RPF2
3: ABCB9	23: PNO1	43: NOB1
4: KCNC2	24: NFX1	44: DDX21
5: FAM120C	25: NOL6	45: YAE1
6: ACTMAP	26: WDR74	46: LTV1
7: KRCC1	27: LONRF1	47: ZNF593
8: HFM1	28: DPY19L1	48: NAA50
9: AAAS	29: NOP56	49: GNL3
10: EEFSEC	30: TSR1	50: EIF4A1
11: MTRFR	31: RRP12	51: ETF1
12: NWD1	32: FTSJ3	52: CCNL1
13: BCAS2	33: NUP153	53: POLR1G
14: UTP4	34: EIF5	54: PPRC1
15: CDC40	35: URB2	55: HABP4
16: XRN1	36: NIFK	56: SMKR1
17: URB1	37: NUP62CL	57: RETREG1
18: BZW2	38: WDR31	58: GPRC5A
19: PELO	39: DDX18	
20: RAE1	40: ZPR1	

Fig. S14. DEG cluster of up- and downregulated genes regulating core cellular machinery program via the production, maturation, and regulation of ribosomes and protein synthesis.

Disease Models & Mechanisms • Supplementary information

Figure – SM15

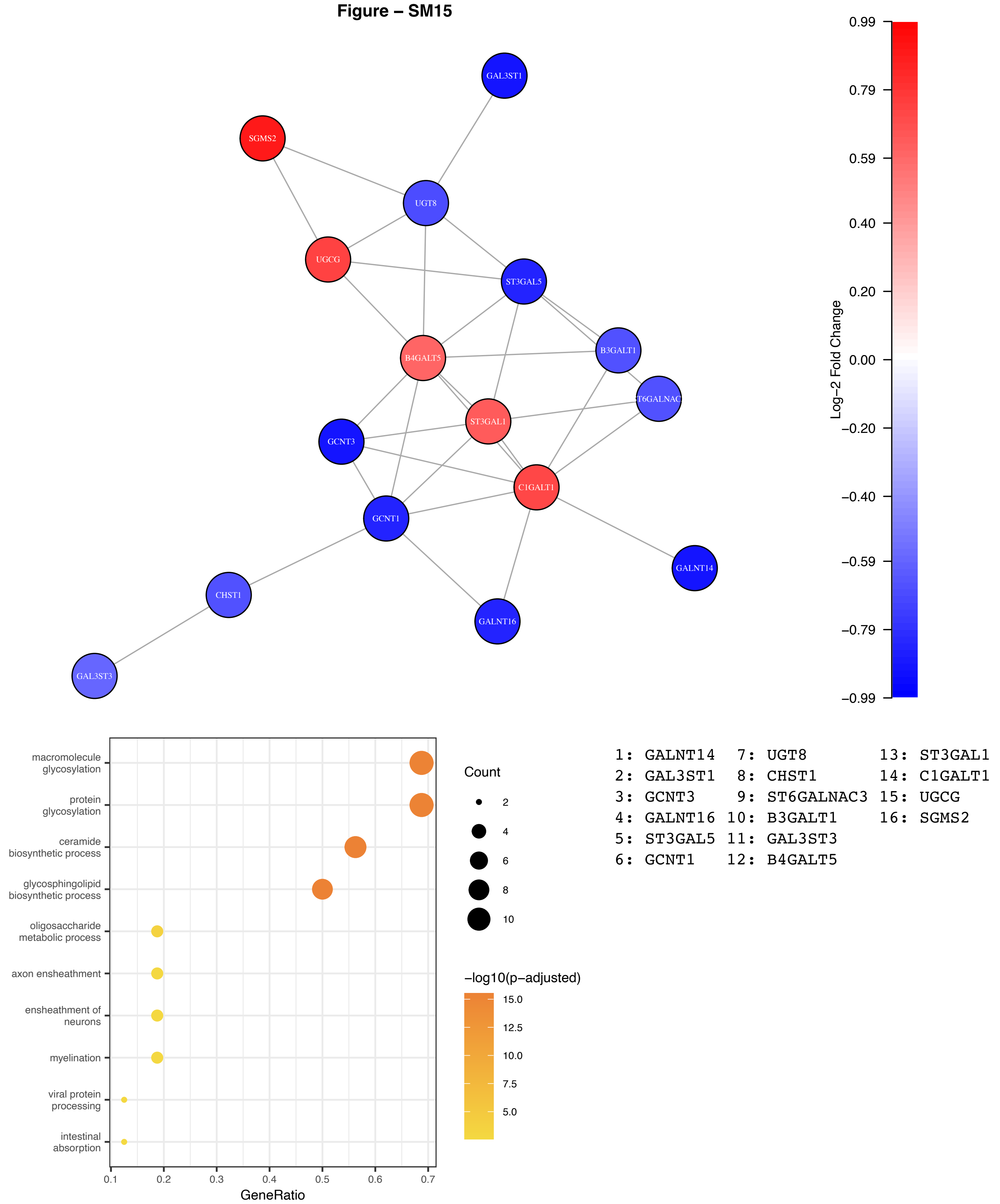

Fig. S15. DEG cluster of up- and downregulated genes regulating cell adhesion, immune recognition, signaling, development, and tissue organization through regulation of glycosylation, glycolipid biosynthesis, and glycoconjugate remodeling.

Disease Models & Mechanisms • Supplementary information

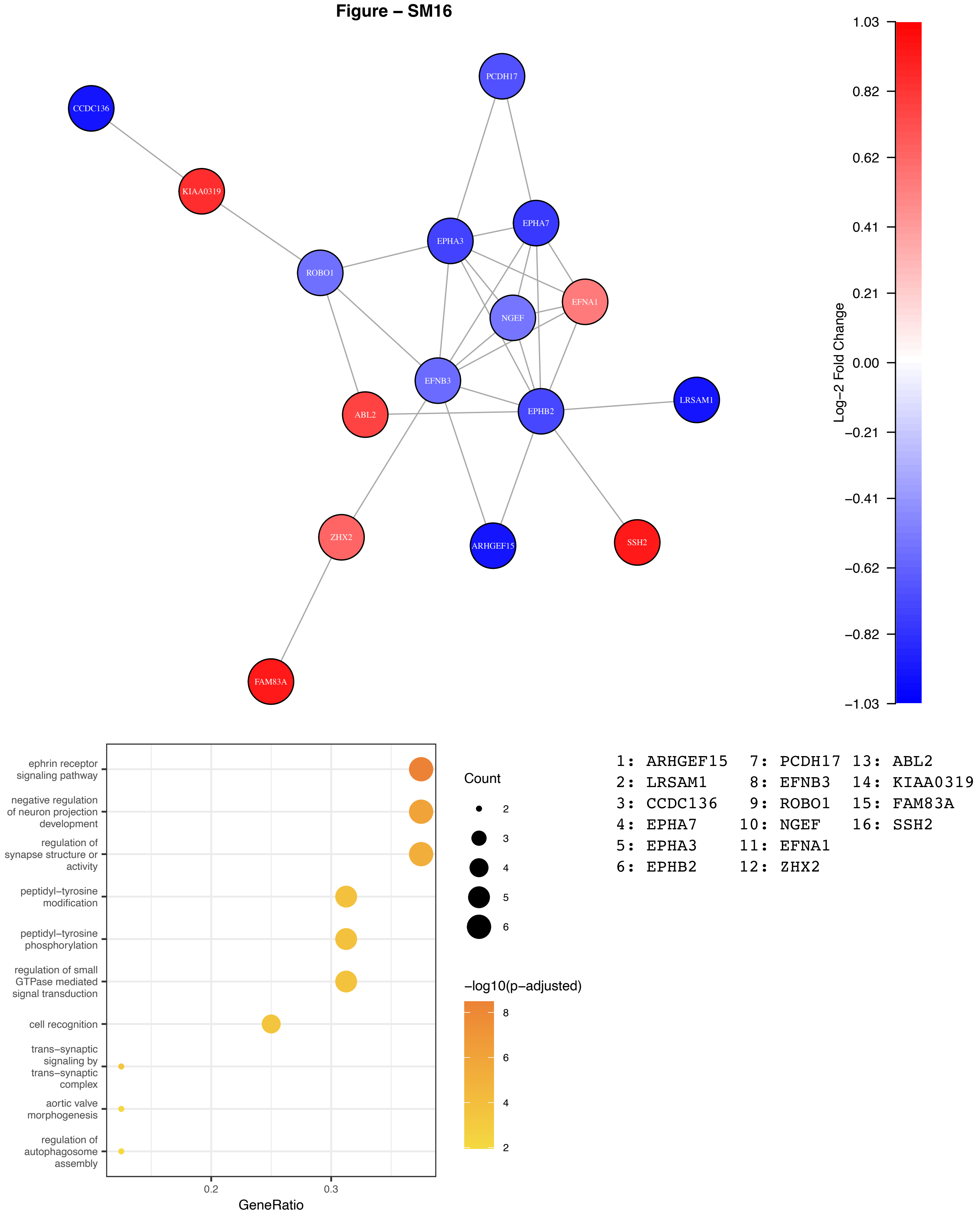

Fig. S16. DEG cluster of up- and downregulated genes regulating cell guidance, receptor-mediated signaling module for cell adhesion, and cytoskeletal remodeling.

Figure – SM17

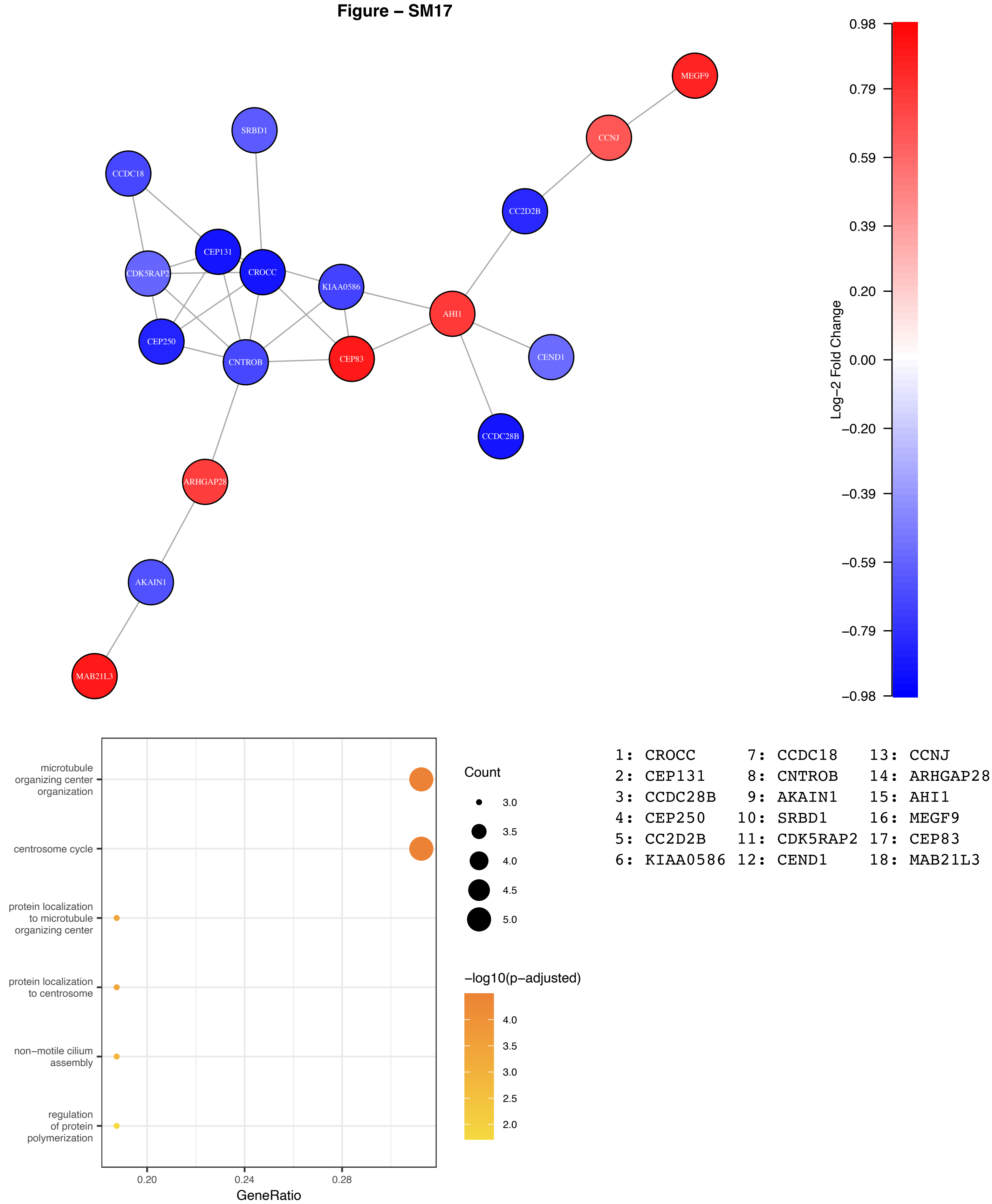

Fig. S17. DEG cluster of up- and downregulated genes regulating primary cilium, centrosome, and microtubule-organizing center (MTOC) module for maintenance of cellular architecture machinery and specialized cellular structures.

Figure – SM18

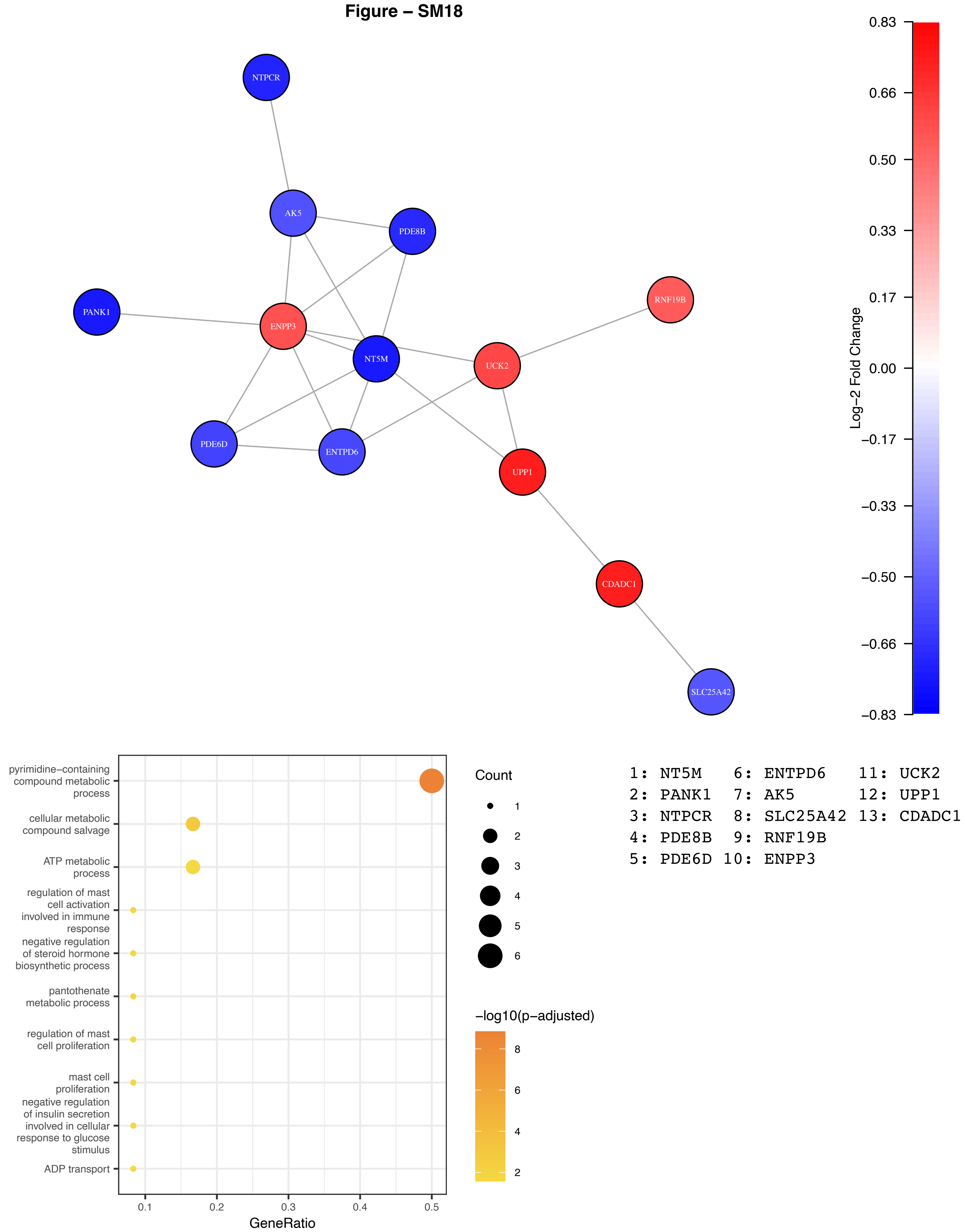

Fig. S18. DEG cluster of up- and downregulated genes regulating the maintenance and recycling of nucleotide pools, coupled with cAMP/cGMP signaling regulation and mitochondrial nucleotide homeostasis.

Disease Models & Mechanisms • Supplementary information

Figure – SM19

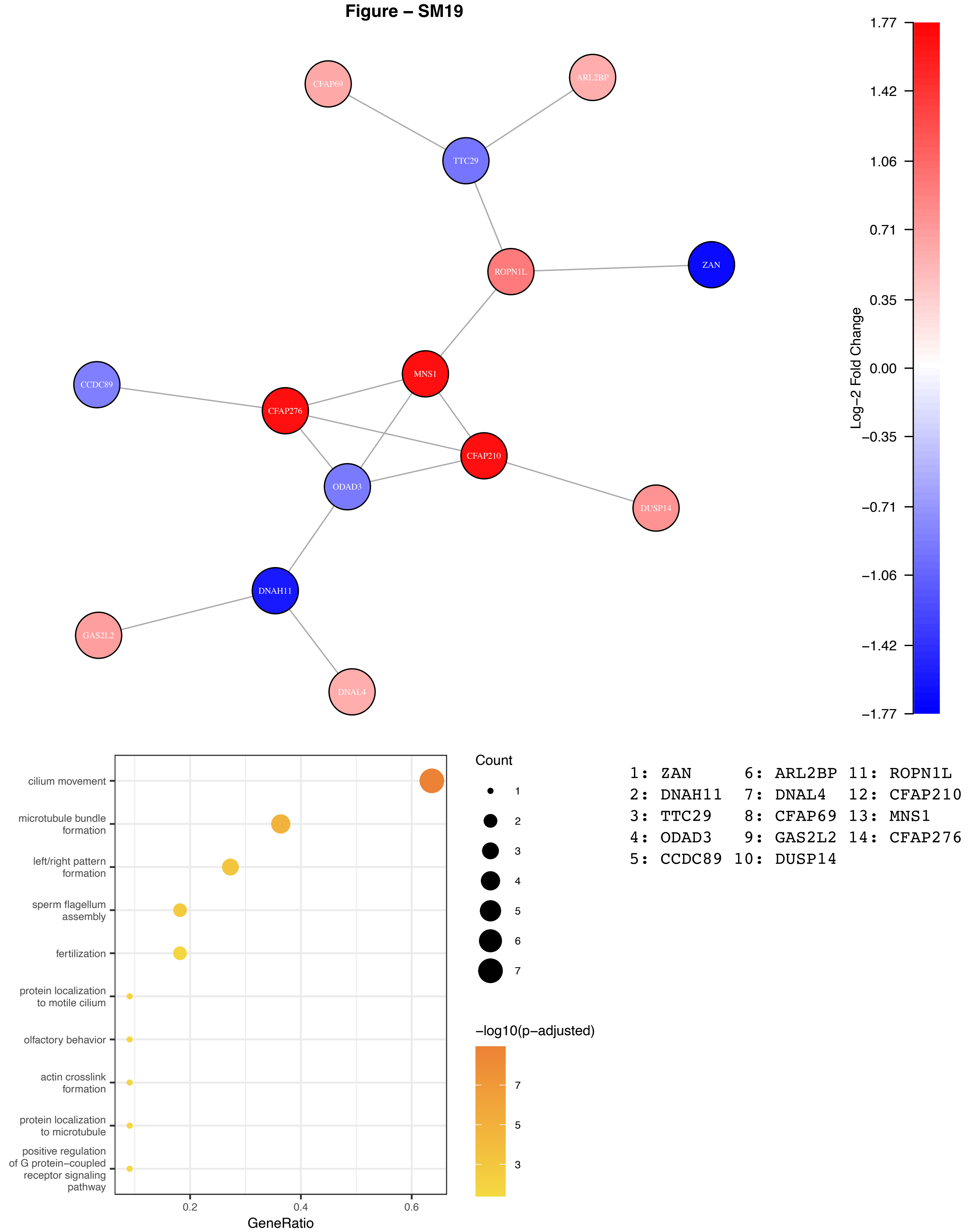

Fig. S19. DEG cluster of up- and downregulated genes regulating microtubular structures facilitation movement, including motile cilia / flagellar axoneme assembly and ciliary beating modules.

Disease Models & Mechanisms • Supplementary information
